# Genome-Wide Analysis to Identify Pathways Affecting Telomere-Initiated Senescence in Budding Yeast

**DOI:** 10.1534/g3.111.000216

**Published:** 2011-08-01

**Authors:** Hsin-Yu Chang, Conor Lawless, Stephen G. Addinall, Sarah Oexle, Morgan Taschuk, Anil Wipat, Darren J. Wilkinson, David Lydall

**Affiliations:** * Institute for Cell and Molecular Biosciences, Medical School, Newcastle University, Framlington Place, Newcastle upon Tyne, NE2 4HH, United Kingdom; † School of Computing Science and ^‡^School of Mathematics and Statistics, Newcastle University, Newcastle upon Tyne, NE1 7RU, United Kingdom; § Centre for Integrated Systems Biology of Ageing and Nutrition, Institute for Ageing and Health, Newcastle University, Newcastle upon Tyne, NE4 5PL, United Kingdom

**Keywords:** telomere, *Saccharomyces cerevisiae*, senescence, crisis

## Abstract

In telomerase-deficient yeast cells, like equivalent mammalian cells, telomeres shorten over many generations until a period of senescence/crisis is reached. After this, a small fraction of cells can escape senescence, principally using recombination-dependent mechanisms. To investigate the pathways that affect entry into and recovery from telomere-driven senescence, we combined a gene deletion disrupting telomerase (*est1Δ*) with the systematic yeast deletion collection and measured senescence characteristics in high-throughput assays. As expected, the vast majority of gene deletions showed no strong effects on entry into/exit from senescence. However, around 200 gene deletions behaving similarly to a *rad52Δest1Δ* archetype (*rad52Δ* affects homologous recombination) accelerated entry into senescence, and such cells often could not recover growth. A smaller number of strains similar to a *rif1Δest1Δ* archetype (*rif1Δ* affects proteins that bind telomeres) accelerated entry into senescence but also accelerated recovery from senescence. Our genome-wide analysis identifies genes that affect entry into and/or exit from telomere-initiated senescence and will be of interest to those studying telomere biology, replicative senescence, cancer, and ageing. Our dataset is complementary to other high-throughput studies relevant to telomere biology, genetic stability, and DNA damage responses.

In most eukaryotes, including mammals and yeasts, telomerase helps maintain telomeric DNA and solve the chromosome end-replication problem (Gomes *et al.* 2010). Telomeric DNA recruits a number of different telomere-binding proteins to make functional telomeres. Dysfunctional telomeres can stimulate DNA damage responses, leading to genetic instability, cell senescence, or apoptosis (Jain and Cooper 2010; Lydall 2009; Stewart and Weinberg 2006).

In most human somatic cells, there is insufficient telomerase to maintain telomere length indefinitely through cell divisions, and consequently, telomeres shorten and ultimately become nonfunctional. Telomere shortening is thought to be a double-edged sword in metazoan cells (Gomes *et al.* 2010; Stewart and Weinberg 2006). On one hand, the limited lifespan of functional telomeres limits cell divisions and therefore protects against uncontrolled cell division in cancer (Artandi and Depinho 2009; Lin *et al.* 2010). On the other hand, limiting cell division prevents tissue renewal and repair and thus contributes to ageing.

Telomere structure and function need to be maintained in cells with unlimited proliferative potential. In the majority of cancers, structure and function are preserved via the expression of high levels of telomerase, permitting unlimited and uncontrolled cell division. However, in around 15% of cancers, telomeres are maintained by alternative lengthening of telomeres (ALT) mechanisms (Henson *et al.* 2002). Therefore a fuller understanding of the pathways allowing eukaryotic cells to tolerate dysfunctional telomeres or to maintain functional telomeres, either in the presence or absence of telomerase, is critical for understanding processes underlying carcinogenesis and aging.

In budding yeast, telomerase is constitutively expressed and telomere length is stable. However, telomerase can be deleted, giving yeast cells a life-cycle much like mammalian somatic cells; their telomeres shorten, they enter into a period of senescence/crisis, and at low rates, the cells recover from senescence using telomerase-independent mechanisms to maintain telomere length and function (Lundblad and Blackburn 1993). Telomerase-deficient yeast cells are therefore an excellent model system to study the pathways that allow eukaryotic cells to tolerate dysfunctional telomeres or to maintain telomeres in the absence of telomerase, and are thus useful for studying pathways relevant to human carcinogenesis. Telomerase-deficient yeast cells typically survive by using recombination-dependent mechanisms to maintain telomeres and are a model for ALT pathways in human cells (Lendvay *et al.* 1996; Lundblad and Blackburn 1993). However, recombination-independent mechanisms for chromosome end protection also occur in budding and fission yeasts (Jain *et al.* 2010; Lee *et al.* 2008; Maringele and Lydall 2004b).

Although a number of yeast genes, principally genes affecting homologous recombination, have been identified that affect entry/exit from telomere driven senescence (Abdallah *et al.* 2009; Azam *et al.* 2006; Chang *et al.* 2011; Enomoto *et al.* 2002; Enomoto *et al.* 2004; Ijpma and Greider 2003; Le *et al.* 1999; Lee *et al.* 2007; Lydeard *et al.* 2007; Maringele and Lydall 2004a; Mceachern and Haber 2006), so far no genome-wide analysis of these processes has been reported. Here we describe the first genome-wide survey to systematically determine the effects of budding yeast gene deletions on the growth of cells with telomere defects. Through these systematic approaches in budding yeast, we identify many yeast genes that affect telomere-driven senescence, orthologs of which may play important roles during carcinogenesis or aging.

## Materials and Methods

### Growth media, synthetic genetic array, and strains

An *est1::natMX* strain (DLY5026) was combined with the genome-wide knockout collection using SGA as previously described (Addinall *et al.* 2011). W303 genetic background strains were cultured in yeast extract, peptone, dextrose, and adenine (YEPD) media.

### Culture passage

#### Solid passage:

1536 colony solid agar-to-solid agar pinning was performed on a Biomatrix BM3-SC robot (S&P Robotics Inc., Toronto, Canada) using an 0.8 mm diameter pin tool.

#### Liquid passage:

Selected *est1::natMX yfg::G418* double mutants were cherry-picked after SGA. Inoculation from solid to liquid media was performed on the Biomatrix BM3-SC robot using a 96-pin (1 mm diameter) pintool. 96-well liquid culture plates were incubated at 30°C for 22 hr without shaking. A Biomek FX robot (Beckman Coulter Limited, High Wycombe, UK) was used to vortex the cultures to resuspend and then inoculated into new sets of 96-well plates containing 200 µl media in each well. A 96-pin (2 mm diameter) pin tool (V&P Scientific, Inc., San Diego, CA, USA) was used, transferring approximately 3 µl of cell culture at each passage. In a similar procedure, resuspended cell cultures were diluted in 200 µl sterile H_2_O and vortexed before the diluted cultures were spotted onto solid agar plates. Agar plates were incubated for 48 hr at 30°C before being photographed.

#### Culture size estimation:

Solid agar plates were photographed on an spImager (S&P Robotics Inc.) and analyzed using Colonyzer (Lawless *et al.* 2010). Culture size variances were stabilized using a Box-Cox transformation with a power parameter of 0.6 as implemented in the R statistical package (R Development Core Team 2011).

#### Mean density profile similarity:

The similarity of the MDP shape of each strain to that of three example archetypes was estimated using Pearson's correlation coefficient (*r*) between strain MDPs and the MDP of each archetype, using the *cor* function in the R statistical package (R Development Core Team 2011). As well as calculating *r*, absolute differences in MDP were estimated using the root mean square (RMS) difference between profiles. The area under each MDP (representing total strain fitness across all passages) was also estimated as the sum of mean culture size across all 22 passages. Lists ranking the MDP for each strain according to its correlation coefficient were generated, with the strains having MDP shape most similar to the archetype (and the archetype itself) at the top of the list (Figure 4, and supporting information, File S5, File S6, File S7, and File S8). Note that the absolute fitness of a comparator strain does not have to be similar to that of the archetype for their MDPs to correlate well. Two strains, one sick and one healthy, can have similarly shaped MDPs that correlate well. It is for this reason that some of the heat maps in Figure 4 are perceptibly stripy.

#### Fraction of recovered repeats:

Recovered fraction for each genotype was estimated by counting the number of repeated cultures for each strain and calculating the fraction of those repeats which had recovered to a size of 1000 or greater by passage 19 (typically in the range from 0/8 to 8/8). For classes similar to each of the three archetypes, the average recovered fraction of class members was calculated.

### Telomere length detection

Southern hybridization to examine telomere length and structure was performed similarly to that previously described (Maringele and Lydall 2004a). Genomic DNA was extracted, digested with *Xho*I, then run overnight on a 1% agarose gel at 1 V/cm. Southern transfer and detection was performed using DIG-High Prime Labeling and Detection Kit (Roche) and visualized on a FUJI LAS4000. Telomeric Y′-TG probes (pHT128; Tsubouchi and Ogawa 2000) were labelled using DIG-High Prime DNA Labeling and Detction Starter Kit II.

## Results

### Measuring senescence by high-throughput, multipassage assays

**Figure 1:**
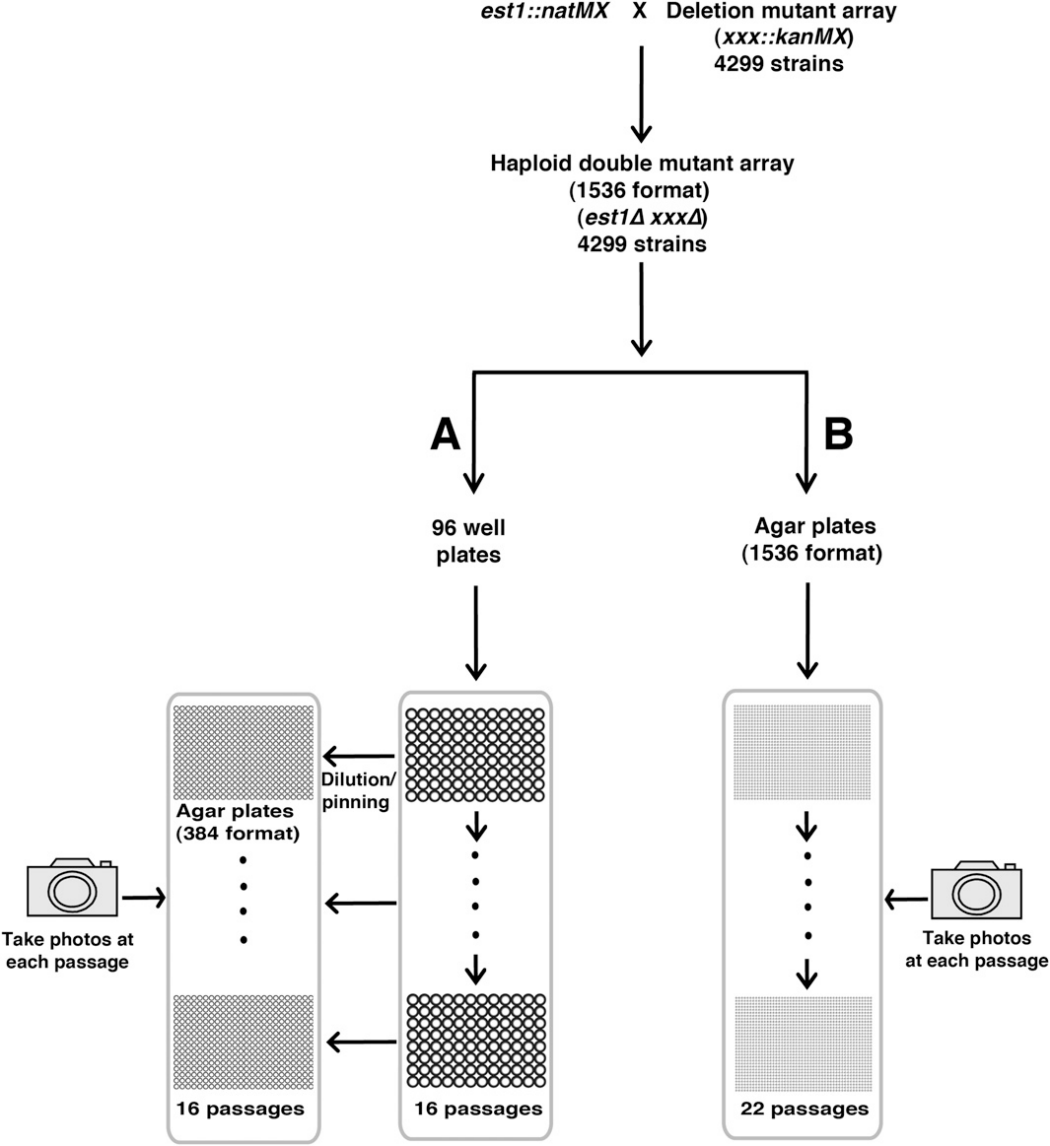
Two methods for genome-wide analysis of entry into and recovery from senescence. An *est1::natMX* strain (DLY5026) was crossed to the deletion mutant collection and double mutants were selected using SGA procedures. (A) Double mutants were passaged in 96-well plate liquid cultures, and fitness was monitored after spotting onto solid agar plates at 384 spot cell density for a total of 16 passages. (B) Cultures were serially pinned at 1536 colonies per plate and photographed for 22 passages.

To identify gene products that affect how telomerase-deficient cells enter into and recover from senescence, we used the synthetic genetic array (SGA) method to combine a systematic genome-wide collection of gene deletions with an *est1∆* mutation defective in telomerase (Costanzo *et al.* 2010). As recovery from senescence is a rare and stochastic process (Lundblad and Blackburn 1993; Nautiyal *et al.* 2002), we compared two high-throughput methods of measuring senescence. These two methods were analogous to low-throughput liquid culture or serial tooth-picking approaches (Lundblad and Blackburn 1993; Maringele and Lydall 2004a). In a liquid procedure we passaged yeast strains in 96-well plates, while in a solid agar procedure we passaged 1536 colonies per plate by pinning directly onto agar (Figure 1). The liquid procedure allowed greater dilution and therefore a greater range of divisions at each passage, but throughput was less efficient than in the pinning procedure. The liquid procedure had a higher risk of culture contamination.

### 96-well liquid senescence assay

For the liquid senescence assay, 96-well plates that contained 60 different *yfg*∆ *est1*∆ genotypes along with 36 independently generated *his3*∆ *est1*∆ “wild type control” strains were inoculated. Cultures in 16 such replicate plates, each with independently generated double mutants, were grown in parallel (Figure S1 shows the plate layout). In combination with *est1*∆, we tested the effects of deleting 27 genes previously reported as affecting telomere length (Askree *et al.* 2004; Gatbonton *et al.* 2006; Shachar *et al.* 2008), 15 affecting DNA replication and repair (Collins *et al.* 2007), and 18 other genes that were randomly chosen controls or of some prior interest (Table S1). The *his3*∆ *est1*∆ strains are suitable “controls” because, although *HIS3* is disrupted with the *KANMX* marker, the cells express sp*HIS5* from the *MATa*-specific promoter and are therefore histidine prototrophs and expected to show no growth defects (Costanzo *et al.* 2010). In total, 16 *yfg*∆ *est1*∆ and 576 *his3*∆ *est1*∆ replicate cultures were examined in these liquid-based experiments.

**Figure 2:**
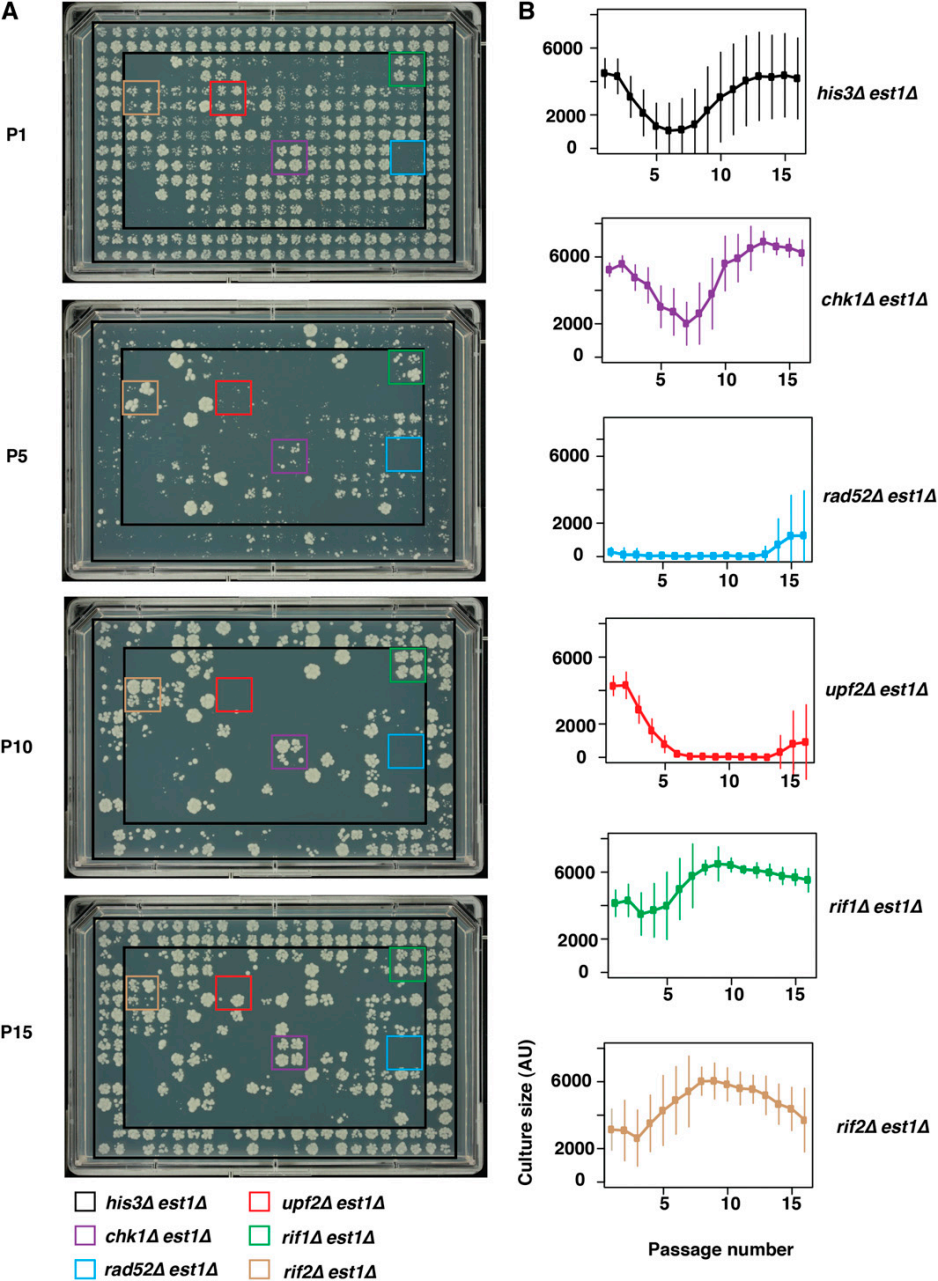
Medium throughput liquid culture based senescence assay. 96-well cultures were spotted at 384 spots per agar plate, and plates photographed and analyzed after 48 hr growth. (A) Example images showing growth at passages 1, 5, 10, and 15. Four independent cultures of five genotypes are illustrated by the colored boxes. (B) Mean density profile (MDP) analysis for six genotypes for 16 passages. Mean culture size and standard deviations are shown. All images can be browsed on our supplementary website (http://research.ncl.ac.uk/colonyzer/ChangSenescence/#images).

To follow growth in liquid, we used the procedure outlined in Figure 1A. Two-hundred microliter (200 μl) cultures were incubated for 22 hr at 30°C without shaking, and then shaken to resuspend the yeast cells. A sample from each culture was then diluted approximately 1:70 into fresh media at each passage using a pin tool. To assess fitness at each passage, a separate sample from each culture was also diluted approximately 1:70 in water and spotted onto agar plates, at 384 spots per plate. Each of four 384-spot plates was incubated for 48 hr and photographed to determine cell density (Figure 2). A 1:70 dilution was a pragmatic choice of dilution in the range used in low-throughput experiments. In low-throughput experiments where we carefully count cell number at each passage and dilute back to 5 × 10^5^ cells per milliliter every day (Figure S3), the dilution ranges from 1:600 for healthy cultures to 1:7 when cells are deeply senescent.

It is clear that the majority of *his3*∆ *est1*∆ strains, situated in the outer two rows/columns of each 384-spot plate, entered into and recovered from senescence over 16 passages (Figure 2). The majority of *his3*∆ *est1*∆ strains grew well at passage 1, by passage 5 they grew very poorly, by passage 10 some had improved, and by passage 15 the majority had returned to a fitness similar to that at passage 1. At passage 15, some *his3*∆ *est1*∆ cultures did not grow at all. This pattern of growth is consistent with the hypothesis that the SGA procedure worked efficiently and that most, but not all, telomerase-deficient *his3*∆ *est1*∆ cultures entered into and recovered from telomere-initiated senescence. Recovery, in particular, was stochastic. To follow the behavior of individual cultures, it is useful to browse through senescent time courses, with each time point shown as a stack, in files (.tiff) from our supplementary data website (http://research.ncl.ac.uk/colonyzer/ChangSenescence/#images).

Because most gene deletions we chose to analyze affect DNA repair or telomere biology, most other strains (*e.g.*, *rad52*∆ *est1*∆ and *upf2*∆ *est1*∆) grew poorly in comparison with *his3*∆ *est1*∆ strains (Figure 2A). *RAD52* is required for recombination-dependent maintenance of telomeres in the absence of telomerase, and as expected, by the first passage the majority of *rad52*∆ *est1*∆ strains were growing poorly and never recovered (Mceachern and Haber 2006). The slight increase in fitness at the end of the time course for *rad52*∆ *est1*∆ strains (Figure 2B) may reflect a *RAD52*-independent mechanism of survivor generation that occurs in this and some other genetic backgrounds (Grandin and Charbonneau 2009; Lebel *et al.* 2009). Interestingly, the effect of *upf2*∆, which affects nonsense-mediated RNA decay, has previously been reported to delay entry into senescence (Enomoto *et al.* 2004), whereas our data suggest that *upf2*∆ accelerates senescence in the *est1*∆ background and that *upf2*∆ *est1*∆ strains only weakly recover (Figure 2).

*chk1*∆ *est1*∆ strains behaved very similarly to *his3*∆ *est1*∆ strains, whereas, interestingly, *rif1*∆ *est1*∆ and *rif2∆est1*∆ strains recovered from senescence more rapidly than the bulk of other strains and the control *his3∆est1*∆ strains. The effects of *rif1*∆ and *rif2*∆ can be seen from the high culture densities for these strains in passages 5 through 10, relative to neighboring cultures (Figure 2A). This is of interest because Rif1 and Rif2 have well-known roles in telomere biology, encoding proteins which bind via Rap1 to telomeric DNA, and are known to affect telomere capping (Addinall *et al.* 2011; Anbalagan *et al.* 2011; Chang *et al.* 2011). Interestingly, and consistent with our results, it was recently reported that *rif1*∆ and *rif2∆* strains enter and recover from senescence more rapidly than other cell types (Chang *et al.* 2011). The positions of all other genes tested by this liquid senescence assay are shown in Figure S1, and one complete series of photographs are shown in Figure S2A. To utilize data from all replicate cultures and to allow quantitative comparison between strains, we constructed mean density profiles (MDP) for all genotypes. MDPs are curves showing the senescence characteristics of each genotype and are constructed after estimating yeast culture cell densities for repeat cultures of different genotypes at each passage from photographs using the image analysis tool Colonyzer (Lawless *et al.* 2010). Six examples of this type of analysis showing the MDP of each are shown in Figure 2B, and all 61 genotypes are shown in File S1. Analysis of 576 independent *his3*∆ *est1*∆ cultures shows that at passage 1 they grew well, with an average fitness of around 4000 units (Figure 2b). As expected, strains became less fit with each passage, reaching a nadir of fitness at around passage 6, before recovering reasonable fitness by the end of the experiment at passage 16. The shape of this curve, combined with the fact that some cultures became sterile, is further evidence that the SGA procedures were efficient and that little contamination by other genotypes (*e.g.*, diploids) or organisms (bacteria) was observed. By passage 16, at the end of the experiment, the mean fitness of *his3*∆ *est1*∆ strains was nearly as high as that at passage 1, which was as expected because of the emergence of recombination-dependent survivors; however, a large variance in cell densities was observed (Figure 2B). This variance at passage 16 was largely due to some cultures being essentially dead (see Figure 2A), presumably because in these particular cultures no senescent cells recovered. Sterile cultures were expected because the majority of cells do not recover from senescence and only a small volume of cell culture (<3 µl) was transferred each time. Variance also began to increase earlier in the experiment, most likely due the stochastic nature of entry into senescence.

As expected from the examples shown in Figure 2A, *chk1*∆ *est1*∆ strains showed a similar MDP to *his3*∆ *est1*∆ strains, although interestingly, there was less variance between repeated *chk1*∆ *est1*∆ cultures and less evidence of loss of vitality with time (Figure 2B, second panel from top). Quantitative analysis of 16 *rad52*∆, *upf2*∆, *rif1*∆, and *rif2∆* strains was consistent with the images.

We used MPDs to visually classify each of the 60 gene deletions into different classes (File S3). These included those that entered senescence rapidly, including *mre11∆*, *xrs2∆*, *rad52∆*, *rad54∆*, *rad55∆*, *rad57∆*, *rtt101∆*, *rtt107∆*, *rtt109∆*, *pol32∆*, *upf1∆*, *upf2∆*, *upf3∆*, *dcc1∆*, *ctf8∆*, *ctf18∆*, *slx5∆*, *slx8∆*, *sgs1∆*, *rad27∆*, *mms1∆*, *mms22∆*, *cdc73∆*, *rtf1∆*, *rfm1∆*, *sum1∆*, *kem1∆*, *rrp8∆*, *asf1∆*, *tel1∆*, *elg1∆*, *hmo1∆*, *ede1∆*, *ydl118w∆*, *rho4∆*, *spt21∆*, *tsa1∆*, *lea1∆*, and *sla1∆*; a large number that behaved normally, including *exo1∆*, *chk1∆*, *rad9∆*; and a small group of just three genes, *est3∆*, *rif1∆*, and *rif2∆*, consisting of mutants that appeared to enter senescence early and recover early. Table S1 summarizes the behavior of individual genes and whether the results we obtained were consistent with previously published studies. It is clear that our results largely reproduced results previously reported from detailed, high-quality, low-throughput experiments giving us confidence in our data. However, there were some interesting surprises, such as the behavior of *upf1∆*, *upf2∆*, and *upf3∆* mutants that failed to recover from senescence here (Figure S5) but had previously been reported to delay senescence (Enomoto *et al.* 2004).

In summary, the 96-well senescence assay was capable of measuring entry of telomerase-deficient cells into senescence and recovery. However, this procedure was comparatively inefficient and not readily scalable to performing a genome-wide analysis of genes that affect entry into/exit from senescence. Therefore, we also tested a much more parallel and efficient solid pinning procedure, discussed next.

### 1536-pin solid-agar senescence assay

**Figure 3:**
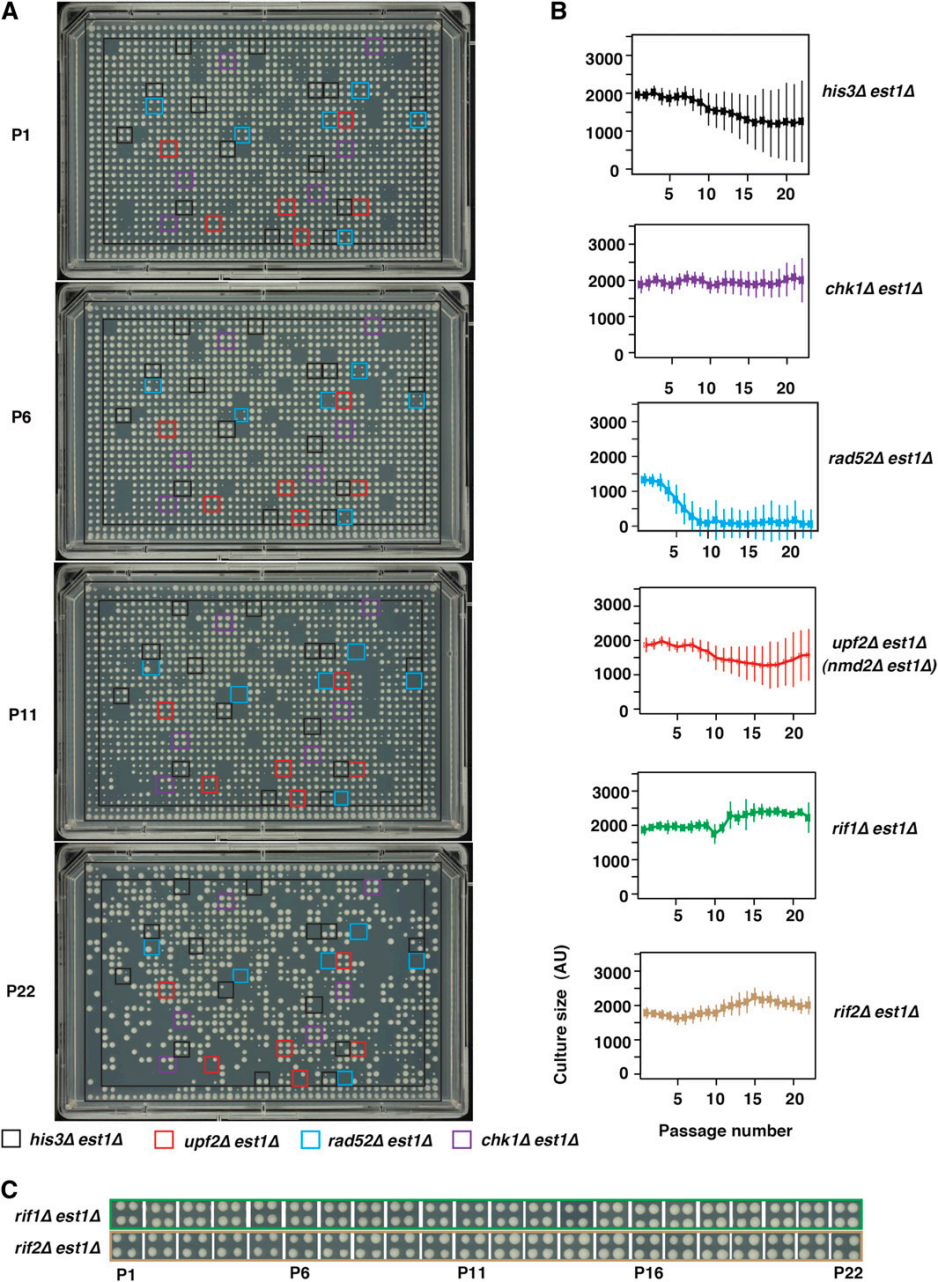
High-throughput, solid-culture-based senescence assay. 1536 parallel colonies were transferred from agar plates, photographed, and analyzed. (A) Example images showing culture size at passages 1, 6, 11, and 22. Representative sets of four independent cultures of four genotypes are indicated by colored boxes. Plate number 15 is presented, which has 24 independently generated double mutant cultures per genotype, whereas plates 1 to 14 only have 4 independent cultures per strain. (B) Mean density profiles (MDP) for six genotypes for 22 passages. Mean culture size and standard deviations are shown. (C) Examples of *rif1∆* and *rif2∆* strain growth during passage. All images can be browsed on our supplementary website (http://research.ncl.ac.uk/colonyzer/ChangSenescence/#images).

By pinning parallel colonies rather than growing liquid cultures, we increased throughput 36-fold. In pinning experiments, we grew a minimum of 8, sometimes 56 (for those on plate 15, which contained 24 extra copies of a small number of gene deletions), and in the case of *his3∆*, 144 independently generated double mutant cultures for a total of 22 passages. Comparing cell density estimates immediately after inoculation with estimates in stationary phase for robustly growing, 1536-format, pinned cultures, we estimate that the maximum dilution achievable at each passage with this method is 16-fold. It is clear that, as cultures were passaged, the fraction of viable cultures decreased, suggesting cells entered into senescence (Figure 3A). In some cultures, culture size decreased to a minimum and then increased (with increasing passage), suggesting that these cultures recovered from senescence. Figure 3A shows plate 15 that, in most cases, contains 24 independently generated *yfg1∆ est1∆* double mutants and numerous *his3∆* strains. Overlaid, colored boxes highlight the positions of *his3∆*, *upf2∆*, *rad52∆*, and *chk1∆* strains, allowing a visual assessment of reproducibility between cultures. Figure S2 compares the growth of cells in the 1536 colony format with the lower throughput, 384-spot format across several passages. All senescent time course photographs, with each time point shown as a stack in files (.tiff), are in our supplementary data website (http://research.ncl.ac.uk/colonyzer/ChangSenescence/#images).

If strains had entered into and recovered from senescence, then telomere structure should alter, showing Type I– and Type II–type structures typical of cells using recombination-dependent ALT mechanisms to maintain telomeres (Lundblad and Blackburn 1993; Teng and Zakian 1999). Importantly, Southern blots showed that the telomere structures of strains that had been passaged on solid plates had indeed altered, forming typical Type I or Type II survivors (Figure S4). In addition many of the DNA repair mutants known to be defective in recovery from senescence did not recover from senescence in these highly parallel experiments (*e.g.*, *rad52∆est1*∆, Figure 3A). These two observations strongly suggest that most, if not all, *est1*∆ cells that were remaining after 22 passages had entered into and recovered from senescence and, therefore, that the high-throughput 1536 solid pinning approach is a useful method for following the senescence process.

We generated MDPs of all 4299 genotypes over 22 passages, and plots of these profiles together with culture size standard deviations are available (File S2). It is interesting to compare the solid pinning data (File S2) with that for the 60 strains also analyzed in liquid culture, 6 of which are shown in Figure 3B, and the remainder of which are shown in File S2. In contrast to the pattern observed in liquid cultures, in pinning experiments evidence of a period of poor growth followed by recovery for many strains was less striking (*e.g.*, MDPs for *his3∆est1∆* and *chk1∆est1∆* strains, Figure 3B). However, it is notable that the variance in fitness increased with passage for each of these strains, more so for *his3∆est1∆* than for *chk1∆est1∆* strains, and that *his3∆est1∆* strains showed an overall decline in fitness that plateaued around passage 15. We believe that the lack of clear decline and recovery in the solid experiments is because of the low dilution at each passage (16-fold in solid *vs.* 70-fold in liquid).

*rad52∆est1∆* strains entered senescence and failed to recover, as expected, but interestingly showed different growth patterns in solid *vs.* liquid plates. At passage 1, the fitness of *rad52∆est1∆* strains (1400 units) was clearly less than the fitness of *est1∆chk1∆* and *est1∆his3∆* strains (2000 units). This most likely is due the low fitness induced by deletion of *RAD52* itself. With time, the fitness of *rad52∆est1∆* declined until, by passage 10, cultures were very small and did not really recover. The rate of entry of *rad52∆est1∆* cultures into senescence was much slower than that observed in liquid cultures, consistent with cultures being diluted less on solid agar compared with liquid cultures and, therefore, having less potential to divide.

Curiously, the density profiles of *upf2∆est1∆* strains on solid pins seemed to be more similar to *his3∆est1∆* strains than to *rad52∆est1∆* strains. Conversely, in liquid cultures, *upf2∆est1∆* strains are more similar to *rad52∆est1∆* strains (compare the density profiles in Figures 2B and 3B). We are unsure of the reason for this difference, but it seems to be reproducible as the effects of *upf1∆*, *upf2∆*, *and upf3∆* mirror each other in both solid and liquid cultures (Figure S5). Disrupting the nonsense-mediated RNA decay pathway affects many different transcripts and, hence, cellular processes, including those relevant to telomere biology (Addinall *et al.* 2011; Enomoto *et al.* 2004). Thus the explanation for the differences between solid and liquid cultures may be as trivial as the *upf∆* strains forming cultures more easily from dense inoculum after pinning on solid agar than from the dilute inoculum used in liquid cultures. Finally *rif1∆est1∆* and *rif2∆est1*∆ mutants essentially behaved as expected given the liquid culture results and, if anything, improved fitness with time (Figure 3B).

### Classifying senescence curves: three archetypes

**Figure 4:**
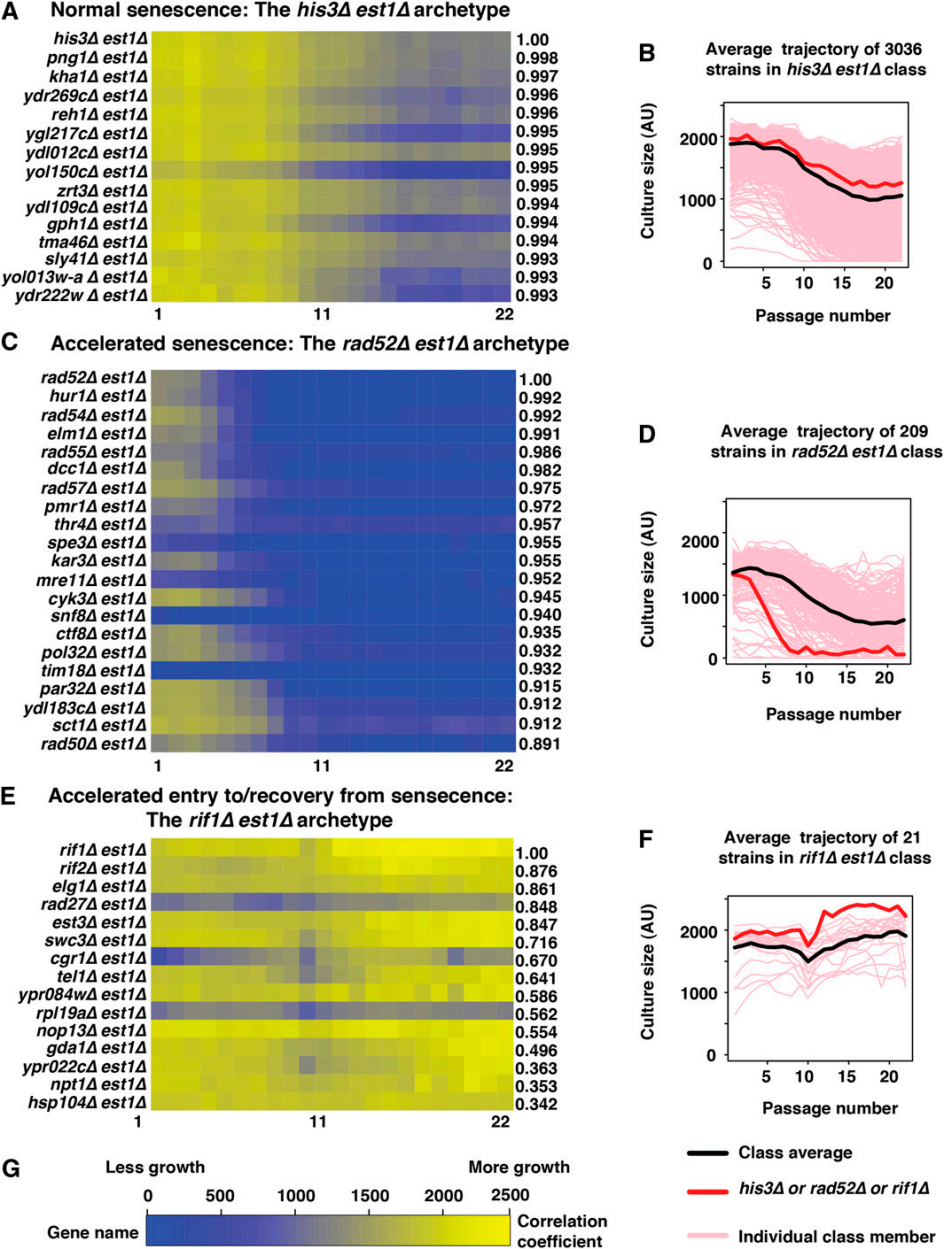
Three senescence profile archetypes. Mean density profiles (MDP) were estimated for each of 4300 gene deletions combined with *est1∆*. Pearson’s correlation coefficient (*r*) was calculated for each mutation compared with *his3∆*, *rad52∆*, and *rif1∆* mutations, representing three distinct senescence curve archetypes. (A) A heat map showing the MDP of the mutations having the 14 highest correlations with that of *his3∆ est1∆*. (B) All 3035 MDPs (pink) correlating strongly (*r* > 0.85) are shown along with the MDP of the archetype *his3∆ est1∆* (red), and the mean curve of the 3036 strongly correlating MDPs (black). (C) A heat map showing the MDP of the mutations having the 20 highest correlations with that of *rad52∆ est1∆*. To construct this group, we only included strains whose culture size at passage 2 was less than *C^s^ = 1680*, where C^s^ = C^ave^ – 0.5*C^SD^, *C^ave^* is the average of all double mutant culture sizes at passage 2, *C^SD^* is the culture size standard deviation at that passage. (D) All 208 MDPs (pink) correlating strongly (*r* > 0.5, culture size < C^s^) with the MDP of *rad52∆ est1∆* (red), together with the average of the 209 strongly correlating MDPs (black). (E) A heat map showing the MDP of the mutations having the 14 highest correlations with that of *rif1∆ est1∆*. (F) Twenty gene disruptions whose MDPs (pink) correlate best with the MDP of *rif1∆ est1∆* (red), together with the average of the 21 most strongly correlating MDPs (black). (G) Scale for heat maps.

To help classify the behavior of the thousands of different genotypes, we generated MDPs for each of the ∼4300 gene deletion strains (File S4). We classified strains according to the similarity of their MDP to that of three archetypal gene disruptions: *his3∆est1∆*, a neutral control strain (Figure 4A, B and File S5); *rad52∆est1∆*, affecting a typical gene required for recovery from senescence (Figure 4C, D, File S6, and File S7); and *rif1∆est1∆*, showing accelerated entry into and exit from senescence (Figure 4E, F and File S8). Similarity between MDPs was quantified using Pearson’s correlation coefficient (*r*).

### Normal senescence: *his3 est1* archetype

As we expected, the vast majority (more than 3000) of the double mutants can be classified in the category of “normal senescence” (Figure 4A), defined as mutants whose MDP correlates well with that of *his3∆est1∆* strains (*r* > 0.85). There was a general trend of lower density across all passages for these strains (Figure 4B); at late passages, this is essentially due to mean density being estimated from a mixture of strongly growing cultures and sterile or poorly growing cultures (*e.g.*, Figure 3A, P22 Supp Fig S2). In this class, the mean density plateaued around passage 18. In normal senescence strains, on average 50% of cultures recovered growth by passage 19. Interestingly, the gene deletions whose MDP have the strongest anticorrelation with *his3∆est1∆* strains include many genes affecting telomere maintenance, DNA damage response, and cell-cycle control (bottom of File S5). In fact, the five strains whose MDP is least well correlated with those of *his3∆est1∆* (*rif2∆est1∆*, *rad27∆est1∆*, *rif1∆est1∆*, *elg1∆est1∆*, and *est3∆est1∆*) are the strongest examples of the *rif1∆est1∆* archetype, which we define below.

### Accelerated entry into and exit from senescence: *rif1 est1* archetype

Using a strict correlation cut off (*r* > 0.85) leaves only three members of the *rif1∆est1∆* class; however, we can see in Figure 4F that the 20 most highly correlated genes display a distinctive MDP. Deletions in this class appear not only to speed up senescence but also to accelerate the appearance of survivors. For strains in this class, on average 81% of cultures recovered growth by passage 19. Interestingly, *EST3*, *RIF1*, *RIF2*, and *TEL1* are genes that are well known to affect telomere maintenance and Chang *et al.* (2011) reported that *rif1*∆ and *rif2*∆ mutants accelerated entry into and recovery from senescence in *rif1∆est2∆* and *rif2∆est2∆* mutants. The effects of *est3∆* on *est1*∆ mutants that we have observed are not consistent with previous reports (Lendvay *et al.* 1996). However, we have confirmed this phenotype in careful low throughput experiments in the different W303 genetic background (Figure S3). The *tel1∆est1∆* results from previous studies are somewhat conflicting (Abdallah *et al.* 2009; Chan *et al.* 2001; Enomoto *et al.* 2002; Ritchie and Petes 2000, Table S1). Some previous data agree with our results whereas other data do not; it is not clear whether the differences are due to strain background or some other factor, such as experimental design. Other genes in this class, notably *ELG1* encoding a replication factor C (RFC)-type protein and with known telomere-related phenotypes (Smolikov *et al.* 2004), warrant further study.

### No recovery from senescence: *rad52 est1* archetype

**Figure 5:**
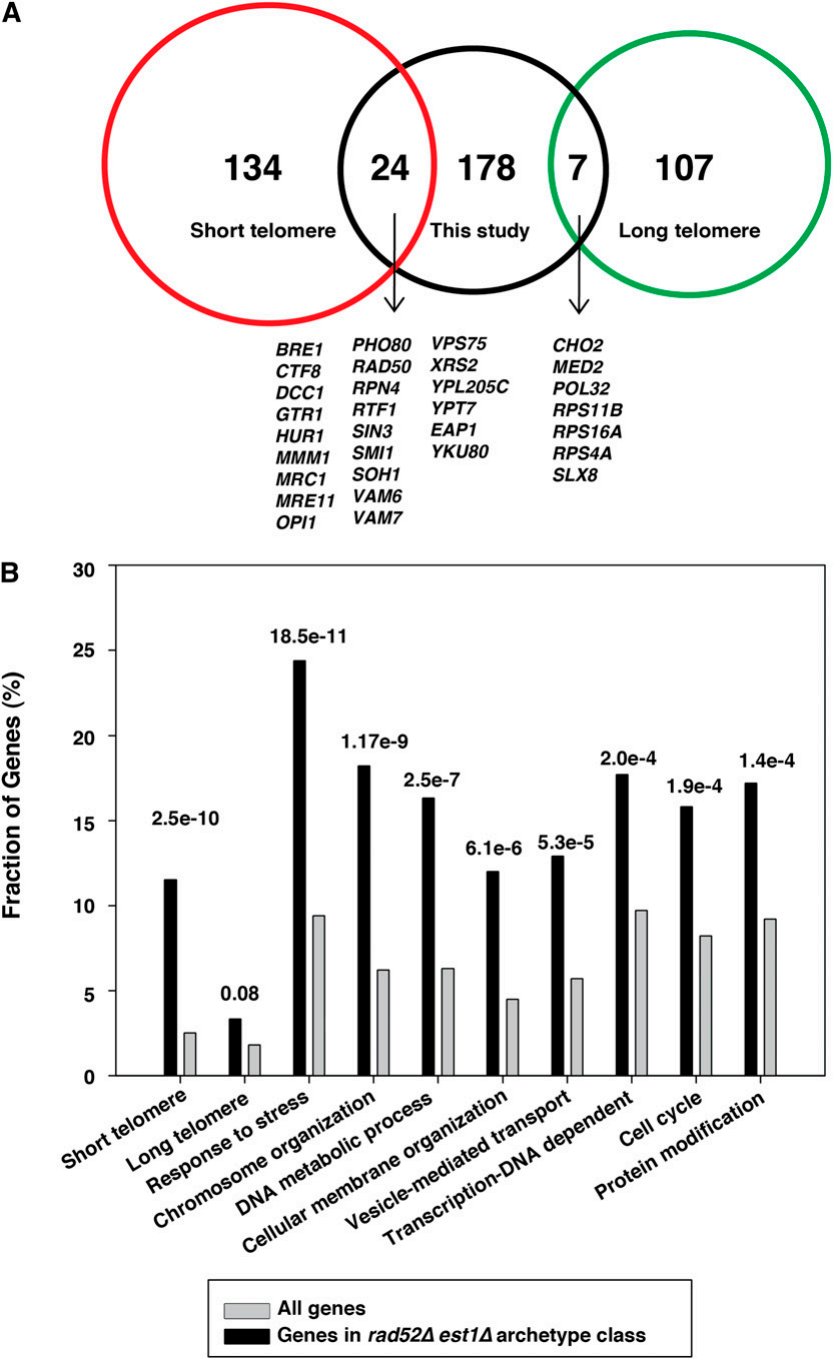
Properties of gene deletions that cause similar effects to *rad52∆* in an *est1∆* background. (A) Venn diagram showing gene deletions reported to result in significantly shorter (red) or longer (green) telomeres (Askree *et al.* 2004; Gatbonton *et al.* 2006; Shachar *et al.* 2008) and those we classified as similar to the *rad52∆ est1∆* archetype (black). (B) Gene ontology (process) analysis demonstrating the *P* values for significance of over representation of terms (both GO terms and classifications from other screens) over represented in the class similar to the *rad52∆ est1∆* archetype.

There were 208 double mutants (File S7) having an MDP that correlated well with that of *rad52∆est1∆* (*r* > 0.5, culture size at passage 2 < 1680, Figure 4C, D). For strains in this class, on average 29% of cultures recovered growth by passage 19. This subset is interesting because it contains many genes required, in principal, for recovery from telomere-initiated senescence, and orthologs of such genes could play critical roles during carcinogenesis, particularly in ALT tumors. Consistent with the role of this gene class at telomeres, there was a strong enrichment of gene deletions previously found to result in short telomeres but not in long telomeres (Askree *et al.* 2004; Gatbonton *et al.* 2006; Shachar *et al.* 2008; Figure 5A). This enrichment is perhaps not surprising because cells with gene deletions inducing short telomeres are likely to enter senescence faster than those with gene deletions that result in normal length or long telomeres.

To further characterize this class, we subjected the genes with MDP similar to the *rad52∆est1∆* archetype to a “gene ontology (GO) yeast process” analysis. Interestingly, we found significant enrichment for genes involved in response to stress, chromosome organization, DNA metabolic processes, cellular membrane organization, vesicle-mediated transport, transcription DNA dependence, cell cycle, and protein modification (Figure 5B). Our results suggest that these genes share many properties and may play an important role in replicative senescence and might do so by affecting several cell processes. Moreover, when subjecting these genes to a GO analysis, we identified many components of known and relevant complexes, for instance, the MRX complex and the Ctf18 RFC–like complex (Table 1). The MRX complex has many known roles at telomeres, and when deletions are combined with deletions in the Ku complex, the double mutants enter senescence like telomerase-deficient strains (Dubois *et al.* 2002; Maringele and Lydall 2004a). However, we are unaware of reports of the MRX complex inhibiting senescence. The Ctf18 complex does have roles at telomeres (Addinall *et al.* 2011; Hiraga *et al.* 2006; Khair *et al.* 2010); however, as far as we are aware, this is the first report showing that deletion of components of this complex accelerates senescence. The mechanisms by which these complexes affect replicative senescence need to be clarified in future studies.

**Table 1:** GO term analysis of *RAD52*-like genes in this study

Macromolecular complex terms	Genes annotated to the term
Ribonucleoprotein complex	*CKB1*, *EAP1,MRPL36*, *MTC1*, *NOP12*, *RPL21B*, *RPL35B*, *RPS11B*, *RPS16A*, *RPS18B*, *RPS4A*, *TEF4*, *TMA19*
Ribosome	*MRPL36*, *MTC1*, *RPL21B*, *RPL35B*, *RPS11B*, *RPS16A*, *RPS18B*, *RPS4A*, *TEF4*, *TMA19*
Chromatin remodeling complex	*ARP6*, *HDA1*, *NHP10*, *SDS3*, *SIN3*, *SWD1*, *SWD3*
Ubiquitin ligase complex	*DIA2*, *MMS1*, *MMS22*, *NPL4*, *SLX5*, *SLX8*, *UBX2*
Preribosome	*CKB1*, *NOP12*, *RPL35B*, *RPS11B*, *RPS16A*, *RPS4A*
SNARE complex	*GOS1*, *TLG2*, *VAM3*, *VAM7*
Elongator holoenzyme complex	*ELP2*, *ELP3*, *ELP4*, *ELP6*
Histone deacetylase complex	*HDA1*, *SDS3*, *SIN3*
Microtubule	*CIK1*, *KAR3*, *SHE1*
Chromatin assembly complex	*ASF1*, *CAC2*, *HTZ1*
Histone acetyltransferase complex	*SGF11*, *SGF29*, *SGF73*
Ctf18 RFC-like complex	*CTF18*, *CTF8*, *DCC1*
MRX complex	*MRE11*, *RAD50*, *XRS2*
DUBm complex	*SGF11*, *SGF73*
Rhp55-Rhp57 complex	*RAD55*, *RAD57*
GSE complex	*GTR1*, *MEH1*
Rpd3L complex	*SDS3*, *SIN3*
Replication fork protection complex	*CTF4*, *MRC1*
HOPS complex	*VAM6*, *VPS41*
Set1C/COMPASS complex	*SWD1*, *SWD3*
Mediator complex	*MED1*, *SOH1*
Vesicle coat	*LST4*, *SEC28*

*RAD52*-like genes in this study were analyzed using SGD gene ontology slim mapper (http://www.yeastgenome.org/cgi-bin/GO/goSlimMapper.pl). “Macromolecular complex terms: Component” GO set was used to determine the list of protein complexes.

### Genes similar to the *rad52* archetype have negative genetic interactions with *yku70* but not *cdc13-1*

**Figure 6:**
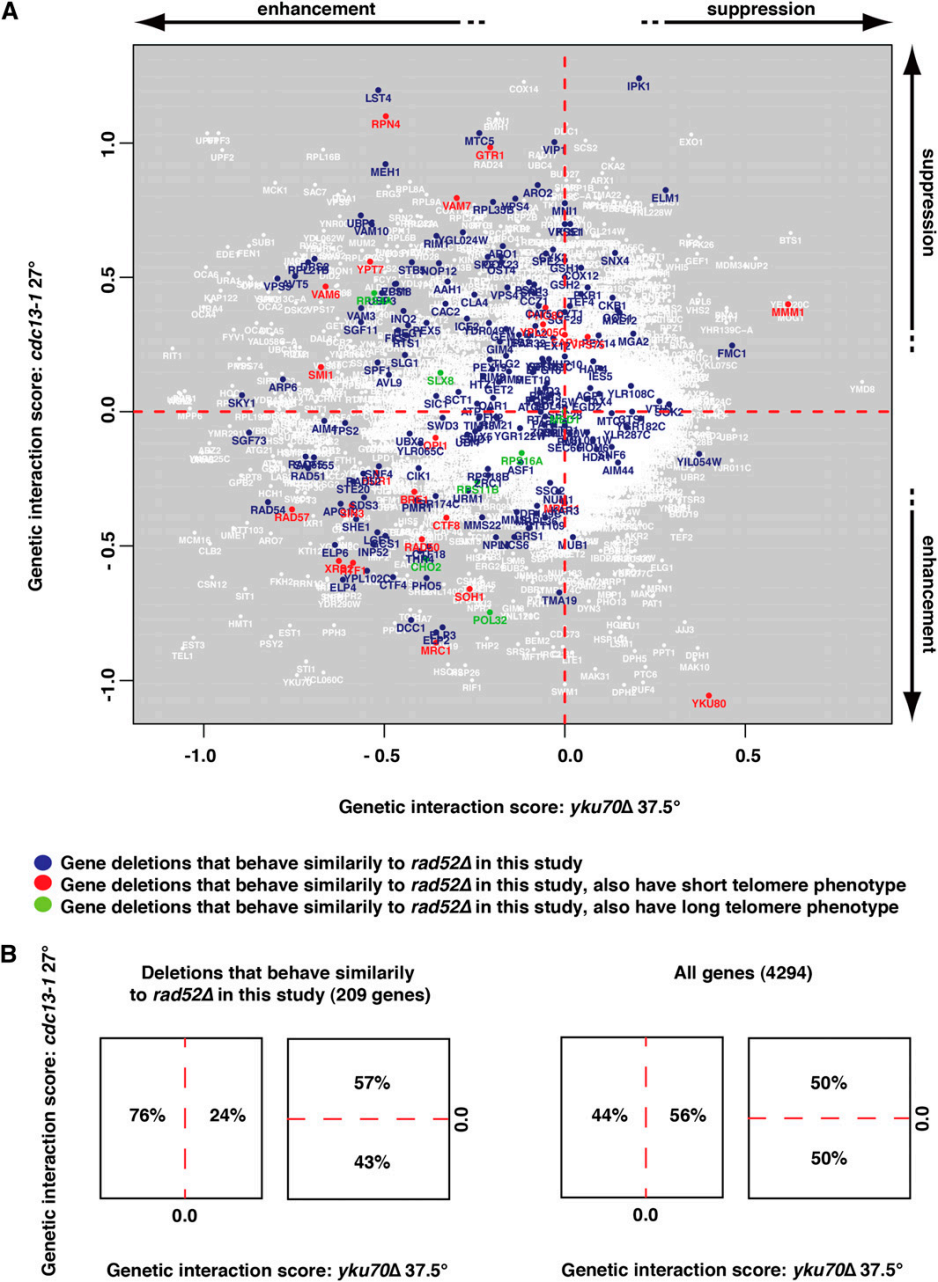
Gene deletions that cause similar effects to *rad52∆* in an *est1∆* background are enriched for deletions that negatively interact with *yku70∆* but not *cdc13-1*. (A) Gene deletions identified in this study as accelerating senescence overlaid on data showing genes that suppress or enhance temperature-sensitive growth defects caused by *cdc13-1* or *yku70∆*. The data points are from Figure 4 in Addinall *et al.* 2011. Genes are highlighted in blue if their mean density profile (MDP) is classed as similar to the *rad52∆ est1∆* archetype, in red if they additionally have a short telomere phenotype, or in green if they additionally have a long telomere phenotype as defined before (Askree *et al.* 2004; Gatbonton *et al.* 2006; Shachar *et al.* 2008) (B) Left: Fraction of strains similar to the *rad52∆ est1∆* archetype found in indicated regions of the genetic interaction profile presented in panel A. Right: Fraction of total gene deletions tested found in indicated regions of the genetic interaction profile presented in panel A.

The high-throughput senesence assays we report here are complementary to other high-throughput studies aimed at understanding telomere or chromosome biology. For example, Addinall *et al.* (2011) performed systematic suppressor/enhancer analyses on yeast strains containing temperature-sensitive telomere capping defects caused by *cdc13-1* or *yku70∆* mutations. All gene deletions were classified as suppressing, enhancing, or showing no strong interaction with each defect, and interaction strengths were quantified. The two datasets were compared in a single plot, and it is interesting to overlay gene deletions that behave like *rad52*∆ *est1∆* in high-throughput senescence assays on this plot (Figure 6A).

Interestingly, most of the gene deletions that accelerated senescence also enhanced *yku70∆* temperature-sensitive growth defects but showed no significant bias toward either positive or negative effects on the fitness of *cdc13-1* mutants. This is shown by the clear left/right but lack of top/bottom bias of such strains in Figure 6. The *yku70∆* mutants have short telomeres, similar to those of telomerase-deficient, pre-senescent cells, whereas *cdc13-1* mutants have telomeres of normal length or slightly longer than normal (Zubko and Lydall 2006). Therefore, a simple explanation for this bias is that the gene deletions highlighted on the left of Figure 6A contribute to the ability of cells with short telomeres to continue through the cell cycle. These data also suggest that *yku70∆* is perhaps a better model for telomere-initiated senescence than *cdc13-1*.

### Genes required for efficient replication fork progression

Global genetic analyses in budding yeast *Saccharomyces cerevisiae* have identified a pathway, including *ASF1*, *RTT109*, *MMS1*, *MMS22*, *RTT101*, and *RTT107*, instrumental to cell survival after replisome stalling (Collins *et al.* 2007; Duro *et al.* 2008; Pan *et al.* 2006; Zaidi *et al.* 2008). Interestingly, it has been proposed that *RAD52* and *MMS1* are essential for pre-senescence growth (Abdallah *et al.* 2009). To further investigate this pathway in telomerase-deficient cells, we examined the behavior of these strains in both our high-throughput and low-throughput screens. We found that not only *RAD52* and *MMS1* but also *ASF1*, *RTT109*, *MMS22*, *RTT101*, and *RTT107* showed an accelerated senescence phenotype when deleted in both screens (Figure 2, Figure 3, Figure S6). These results strongly suggest that replication fork progression plays an important role in replicative senescence. However, our data also suggest that there are many additional pathways/complexes affecting replicative senescence (Figure 5, Table 1). It will be interesting to investigate the roles of these pathways/complexes further.

## Discussion

We report the first genome-wide analysis of the effects of gene deletions on entry into and exit from telomere-initiated senescence in *S. cerevisiae*. We have successfully carried out a medium-throughput, liquid-based senescence screen and a high-throughput, solid pin-based screen. Although the liquid-based screen is a valuable source of data describing the behavior of 60 gene deletions hand-picked for the relevance of their effect on entry into and recovery from senescence, we chose to perform a genome-wide analysis of 4299 genes by using a solid pinning approach because it was much more efficient (36 times the throughput). Inevitably, the high-throughput pinning approach suffers from a lack of resolution compared with the liquid-based approach. Nevertheless, it contains a great deal of valuable quantitative information. We have constructed a high-quality dataset demonstrating senescence profiles for all 4299 gene deletions in the knockout library combined with a telomerase defect that will complement other low- and high-throughput analyses of telomere biology and the DNA damage response.

We chose to classify double deletion mutations based on the similarity of their MDPs to those of archetypal mutations: *his3∆est1∆* (normal), *rad52∆est1∆* (accelerated entry into senescence and failure to recover), and *rif1∆est1∆* (accelerated entry into senescence and recovery). However, other clustering or analysis approaches may also prove informative. For example, we also performed unsupervised hierarchical quality threshold (QT) clustering to identify 23 different MDP classes (Figure S8). The MDPs which we constructed (*e.g.*, File S2 and File S4), together with the images and raw quantifications from which they are derived, are published to allow others to analyze our dataset using different approaches, for example, by searching for strains whose deletions result in MDPs correlating strongly with those of archetypes of specific interest, which could be different to the three presented here.

The *rad52*∆ *est1∆* archetype strain and mutants in this class show poor growth when generated by SGA immediately after germination. Therefore we tested whether additional information is gained from continuing to passage such poor-growing strains. To address this issue, we chose to compare our *rad52∆*-like cluster with genes we identified as growing poorly with *est1∆* at passage 0 in our experiments, and with analogous datasets from large-scale, genome-wide synthetic lethal analysis experiments (Costanzo *et al.* 2010). Figure S7A shows that there is overlap among these three datasets but that they are clearly not identical. The group of strains with MDP similar to that of the *rad52*∆ *est1∆* archetype is distinct from that which we identified as synthetic lethal interactions immediately after SGA (File S10 and File S11).

To compare the relative merits of each dataset, the member strains of each are highlighted in Figure S7C–F on plots similar to Figure 6. Interestingly, the left right/bias we observed for the strains in the *rad52∆est1∆* class (Figure 6 and S7C) is not as pronounced in the other datasets. This strongly suggests that gene deletions we identified in the *rad52∆est1∆* class, based on their behavior with repeated passage, have different properties than those growing poorly at early passages identified by us or in the Costanzo *et al.* (2010) dataset. Therefore we conclude that, at the very least, our high-throughput senescence assays complement the existing datasets, identifying gene deletions with notably different properties.

Our dataset is a rich resource for those working in telomere biology in budding yeast and other eukaryotic organisms. All our data are freely available, and there are numerous provocative and interesting observations worthy of further investigation. For example, the genes behaving like *RAD52* or *RIF1* show very interesting effects on the senescence process in budding yeast. Although some genes in the *RAD52* class, such as *SLX5*, *SLX8*, and *MMS1*, were previously reported to affect the processes of recovery from senescence, we have identified 200 or more genes in this class. Our data also shed new light on the functions of proteins with known roles in telomere biology, such as Elg1 or members of the Ctf18 complex.

We imagine that iterations of our genome-wide approach to understand senescence, perhaps utilizing genome-wide liquid passage approaches, like Figure 2, or “bar code–based” approaches (Hillenmeyer *et al.* 2008), may extend our systematic analysis of gene deletions affecting telomere-driven senescence. It may also be interesting to perform genome-wide screens analyzing the effects of deleting other protein or RNA subunits of telomerase, as we have shown that deleting the *EST3* gene affects the senescence profile of *est1*∆ mutants in a manner analogous to *rif1∆* mutants. It may also be interesting to extend this approach to *S. pombe*, where telomere biology is significantly different from *S. cerevisiae* and genome-wide knockout libraries and SGA approaches are now available (Dixon *et al.* 2008; Roguev *et al.* 2007). It will be more technically challenging to perform analogous studies in mammalian cells.

## Supplementary Material

Supporting Information

## References

[bib1] Abdallah P. Luciano P. Runge K. W. Lisby M. Geli V., 2009 A two-step model for senescence triggered by a single critically short telomere. Nat. Cell Biol. 11: 988–993 19597486 10.1038/ncb1911 PMC4025917

[bib2] Addinall S. G. Holstein E.-M. Lawless C. Yu M. Chapman K., 2011 Quantitative fitness analysis shows that NMD proteins and many other protein complexes suppress or enhance distinct telomere cap defects. PLoS Genet. 7: e1001362 21490951 10.1371/journal.pgen.1001362 PMC3072368

[bib3] Anbalagan S. Bonetti D. Lucchini G. Longhese M. P., 2011 Rif1 supports the function of the CST complex in yeast telomere capping. PLoS Genet. 7: e1002024 21437267 10.1371/journal.pgen.1002024 PMC3060071

[bib4] Artandi S. E. DePinho R. A., 2009 Telomeres and telomerase in cancer. Carcinogenesis 31: 9–18 19887512 10.1093/carcin/bgp268 PMC3003493

[bib5] Askree S. H. Yehuda T. Smolikov S. Gurevich R. Hawk J., 2004 A genome-wide screen for Saccharomyces cerevisiae deletion mutants that affect telomere length. Proc. Natl. Acad. Sci. U S A 101: 8658–8663 15161972 10.1073/pnas.0401263101 PMC423251

[bib6] Azam M. Lee J. Y. Abraham V. Chanoux R. Schoenly K. A., 2006 Evidence that the S.cerevisiae Sgs1 protein facilitates recombinational repair of telomeres during senescence. Nucleic Acids Res. 34: 506–516 16428246 10.1093/nar/gkj452 PMC1342037

[bib7] Chan S. W. Chang J. Prescott J. Blackburn E. H., 2001 Altering telomere structure allows telomerase to act in yeast lacking ATM kinases. Curr. Biol. 11: 1240–1250 11525738 10.1016/s0960-9822(01)00391-8

[bib8] Chang M. Dittmar J. C. Rothstein R., 2011 Long telomeres are preferentially extended during recombination-mediated telomere maintenance. Nat. Struct. Mol. Biol. 18: 451–456 21441915 10.1038/nsmb.2034 PMC3071861

[bib9] Collins S. R. Miller K. M. Maas N. L. Roguev A. Fillingham J., 2007 Functional dissection of protein complexes involved in yeast chromosome biology using a genetic interaction map. Nature 446: 806–810 17314980 10.1038/nature05649

[bib10] Costanzo M. Baryshnikova A. Bellay J. Kim Y. Spear E. D., 2010a The genetic landscape of a cell. Science 327: 425–431 20093466 10.1126/science.1180823 PMC5600254

[bib12] Dixon S. J. Fedyshyn Y. Koh J. L. Prasad T. S. Chahwan C., 2008 Significant conservation of synthetic lethal genetic interaction networks between distantly related eukaryotes. Proc. Natl. Acad. Sci. U S A 105: 16653–16658 18931302 10.1073/pnas.0806261105 PMC2575475

[bib13] DuBois M. L. Haimberger Z. W. McIntosh M. W. Gottschling D. E., 2002 A quantitative assay for telomere protection in Saccharomyces cerevisiae. Genetics 161: 995–1013 12136006 10.1093/genetics/161.3.995 PMC1462171

[bib14] Duro E. Vaisica J. A. Brown G. W. Rouse J., 2008 Budding yeast Mms22 and Mms1 regulate homologous recombination induced by replisome blockage. DNA Repair (Amst.) 7: 811–818 18321796 10.1016/j.dnarep.2008.01.007

[bib15] Enomoto S. Glowczewski L. Berman J., 2002 MEC3, MEC1, and DDC2 are essential components of a telomere checkpoint pathway required for cell cycle arrest during senescence in Saccharomyces cerevisiae. Mol. Biol. Cell 13: 2626–2638 12181334 10.1091/mbc.02-02-0012 PMC117930

[bib16] Enomoto S. Glowczewski L. Lew-Smith J. Berman J. G., 2004 Telomere cap components influence the rate of senescence in telomerase-deficient yeast cells. Mol. Cell. Biol. 24: 837–845 14701754 10.1128/MCB.24.2.837-845.2004 PMC343809

[bib17] Gatbonton T. Imbesi M. Nelson M. Akey J. M. Ruderfer D. M., 2006 Telomere length as a quantitative trait: genome-wide survey and genetic mapping of telomere length-control genes in yeast. PLoS Genet. 2: e35 16552446 10.1371/journal.pgen.0020035 PMC1401499

[bib18] Gomes N. M. Shay J. W. Wright W. E., 2010 Telomere biology in Metazoa. FEBS Lett. 584: 3741–3751 20655915 10.1016/j.febslet.2010.07.031 PMC2928394

[bib19] Grandin N. Charbonneau M., 2009 Telomerase- and RAD52-independent immortalization of budding yeast by an inherited-long-telomere pathway of telomeric repeat amplification. Mol. Cell. Biol. 29: 965–985 19047370 10.1128/MCB.00817-08 PMC2643805

[bib20] Henson J. D. Neumann A. A. Yeager T. R. Reddel R. R., 2002 Alternative lengthening of telomeres in mammalian cells. Oncogene 21: 598–610 11850785 10.1038/sj.onc.1205058

[bib21] Hillenmeyer M. E. Fung E. Wildenhain J. Pierce S. E. Hoon S., 2008 The chemical genomic portrait of yeast: uncovering a phenotype for all genes. Science 320: 362–365 18420932 10.1126/science.1150021 PMC2794835

[bib22] Hiraga S. Robertson E. D. Donaldson A. D., 2006 The Ctf18 RFC-like complex positions yeast telomeres but does not specify their replication time. EMBO J. 25: 1505–1514 16525505 10.1038/sj.emboj.7601038 PMC1440320

[bib23] IJpma A. Greider C. W., 2003 Short telomeres induce a DNA damage response in Saccharomyces cerevisiae. Mol. Biol. Cell 14: 987–1001 12631718 10.1091/mbc.02-04-0057 PMC151574

[bib24] Jain D. Cooper J. P., 2010 Telomeric strategies: means to an end. Annu. Rev. Genet. 44: 243–269 21047259 10.1146/annurev-genet-102108-134841

[bib25] Jain D. Hebden A. K. Nakamura T. M. Miller K. M. Cooper J. P., 2010 HAATI survivors replace canonical telomeres with blocks of generic heterochromatin. Nature 467: 223–227 20829796 10.1038/nature09374

[bib26] Khair L. Chang Y. T. Subramanian L. Russell P. Nakamura T. M., 2010 Roles of the checkpoint sensor clamp Rad9-Rad1-Hus1 (911)-complex and the clamp loaders Rad17-RFC and Ctf18-RFC in Schizosaccharomyces pombe telomere maintenance. Cell Cycle 9: 2237–2248 20505337 10.4161/cc.9.11.11920 PMC3133598

[bib27] Lawless C. Wilkinson D. J. Young A. Addinall S. G. Lydall D. A., 2010 Colonyzer: automated quantification of micro-organism growth characteristics on solid agar. BMC Bioinformatics 11: 287 20509870 10.1186/1471-2105-11-287 PMC2901369

[bib28] Le S. Moore J. K. Haber J. E. Greider C. W., 1999 RAD50 and RAD51 define two pathways that collaborate to maintain telomeres in the absence of telomerase. Genetics 152: 143–152 10224249 10.1093/genetics/152.1.143 PMC1460580

[bib29] Lebel C. Rosonina E. Sealey D. C. Pryde F. Lydall D., 2009 Telomere maintenance and survival in saccharomyces cerevisiae in the absence of telomerase and RAD52. Genetics 182: 671–684 19380905 10.1534/genetics.109.102939 PMC2710150

[bib30] Lee J. Y. Kozak M. Martin J. D. Pennock E. Johnson F. B., 2007 Evidence that a RecQ helicase slows senescence by resolving recombining telomeres. PLoS Biol. 5: 1334–1344 10.1371/journal.pbio.0050160 PMC1885831 17550308

[bib31] Lee J. Y. Mogen J. L. Chavez A. Johnson F. B., 2008 Sgs1 RecQ helicase inhibits survival of Saccharomyces cerevisiae cells lacking telomerase and homologous recombination. J. Biol. Chem. 283: 29847–29858 18757364 10.1074/jbc.M804760200 PMC2573055

[bib32] Lendvay T. S. Morris D. K. Sah J. Balasubramanian B. Lundblad V., 1996 Senescence mutants of Saccharomyces cerevisiae with a defect in telomere replication identify three additional EST genes. Genetics 144: 1399–1412 8978029 10.1093/genetics/144.4.1399 PMC1207693

[bib33] Lin T. T. Letsolo B. T. Jones R. E. Rowson J. Pratt G., 2010 Telomere dysfunction and fusion during the progression of chronic lymphocytic leukemia: evidence for a telomere crisis. Blood 116: 1899–1907 20538793 10.1182/blood-2010-02-272104

[bib34] Lundblad V. Blackburn E. H., 1993 An alternative pathway for yeast telomere maintenance rescues est1^−^ senescence. Cell 73: 347–360 8477448 10.1016/0092-8674(93)90234-h

[bib35] Lydall D., 2009 Taming the tiger by the tail: modulation of DNA damage responses by telomeres. EMBO J. 28: 2174–2187 19629039 10.1038/emboj.2009.176 PMC2722249

[bib36] Lydeard J. R. Jain S. Yamaguchi M. Haber J. E., 2007 Break-induced replication and telomerase-independent telomere maintenance require Pol32. Nature 448: 820–823 17671506 10.1038/nature06047

[bib37] Maringele L. Lydall D., 2004a EXO1 plays a role in generating type I and type II survivors in budding yeast. Genetics 166: 1641–1649 15126386 10.1534/genetics.166.4.1641 PMC1470825

[bib38] Maringele L. Lydall D., 2004b Telomerase- and recombination-independent immortalization of budding yeast. Genes Dev. 18: 2663–2675 15489288 10.1101/gad.316504 PMC525546

[bib39] McEachern M. J. Haber J. E., 2006 Break-induced replication and recombinational telomere elongation in yeast. Annu. Rev. Biochem. 75: 111–135 16756487 10.1146/annurev.biochem.74.082803.133234

[bib50] Nautiyal S. DeRisi J. L. Blackburn E. H., 2002 The genome-wide expression response to telomerase deletion in Saccharomyces cerevisiae. Proc. Natl. Acad. Sci. USA 14: 9316–9321 Erratum in Proc. Natl. Acad. Sci. USA 35:13100 12084816 10.1073/pnas.142162499 PMC123138

[bib40] Pan X. Ye P. Yuan D. S. Wang X. Bader J. S., 2006 A DNA integrity network in the yeast Saccharomyces cerevisiae. Cell 124: 1069–1081 16487579 10.1016/j.cell.2005.12.036

[bib41] R Development Core Team, 2011 R: A Language and Environment for Statistical Computing. R Foundation for Statistical Computing, Vienna, Austria

[bib42] Ritchie K. B. Petes T. D., 2000 The Mre11p/Rad50p/Xrs2p complex and the Tel1p function in a single pathway for telomere maintenance in yeast. Genetics 155: 475–479 10790418 10.1093/genetics/155.1.475 PMC1461057

[bib43] Roguev A. Wiren M. Weissman J. S. Krogan N. J., 2007 High-throughput genetic interaction mapping in the fission yeast Schizosaccharomyces pombe. Nat. Methods 4: 861–866 17893680 10.1038/nmeth1098

[bib44] Shachar R. Ungar L. Kupiec M. Ruppin E. Sharan R., 2008 A systems-level approach to mapping the telomere length maintenance gene circuitry. Mol. Syst. Biol. 4: 172 18319724 10.1038/msb.2008.13 PMC2290934

[bib45] Smolikov S. Mazor Y. Krauskopf A., 2004 ELG1, a regulator of genome stability, has a role in telomere length regulation and in silencing. Proc. Natl. Acad. Sci. U S A 101: 1656–1661 14745004 10.1073/pnas.0307796100 PMC341813

[bib46] Stewart S. A. Weinberg R. A., 2006 Telomeres: cancer to human aging. Annu. Rev. Cell Dev. Biol. 22: 531–557 16824017 10.1146/annurev.cellbio.22.010305.104518

[bib47] Teng S. C. Zakian V. A., 1999 Telomere-telomere recombination is an efficient bypass pathway for telomere maintenance in Saccharomyces cerevisiae. Mol. Cell. Biol. 19: 8083–8093 10567534 10.1128/mcb.19.12.8083 PMC84893

[bib51] Tsubouchi H. Ogawa H., 2000 Exo1 roles for repair of DNA double-strand breaks and meiotic crossing over in Saccharomyces cerevisiae. Mol. Biol. Cell 11: 2221–2233 10888664 10.1091/mbc.11.7.2221 PMC14915

[bib48] Zaidi I. W. Rabut G. Poveda A. Scheel H. Malmstrom J., 2008 Rtt101 and Mms1 in budding yeast form a CUL4(DDB1)-like ubiquitin ligase that promotes replication through damaged DNA. EMBO Rep. 9: 1034–1040 18704118 10.1038/embor.2008.155 PMC2572122

[bib49] Zubko M. K. Lydall D., 2006 Linear chromosome maintenance in the absence of essential telomere-capping proteins. Nat. Cell Biol. 8: 734–740 16767084 10.1038/ncb1428

