# Supplementary material for: Genome-Wide Analysis to Identify Pathways Affecting Telomere-Initiated Senescence in Budding Yeast
Source: G3 (Bethesda). 2011 Aug 1;1(3):197–208. doi: 10.1534/g3.111.000216 (PMC3276134; doi:10.1534/g3.111.000216)
Supplement: Supporting Information [file supp_1_3_197__index.html]

Supporting Information 

# Genome-Wide Analysis to Identify Pathways Affecting Telomere-Initiated Senescence in Budding Yeast

## Supporting Information for Chang *et al.*, 2011

**Files in this Data Supplement:**

- Supporting Information - Figures S1-S8, Files S1-S13, and Tables S1 and S2 (PDF, 7.7 MB)
- Figure S1 - 60 *yfg∆ est1∆* strains arrayed in quadruplicate on a 384 format plate (PDF, 2.3 MB)
- Figure S2 - Example photographs from passaged cultures (PDF, 1.7 MB)
- Figure S3 - Low through-put senescence experiment in the W303 genetic background (PDF, 100 KB)
- Figure S4 - Survivors were produced by passage 22 in the solid procedure (PDF, 1.2 MB)
- Figure S5 - Nonsense mediated decay genes had consistent MDPs in both screens (PDF, 92 KB)
- Figure S6 - Genes affecting replication fork progression had an accelerated senescence phenotype when deleted in the est1∆ background (PDF, 208 KB)
- Figure S7 - Repeated passage of cultures provides useful information about telomere-dependent senescence (PDF, 1.3 MB)
- Figure S8 - Unsupervised hierarchical QT clustering identifies 23 different MDP classes (PDF, 600 KB)
- Files S1-S13 - All supporting files listed in this PDF (together with raw images and initial culture size quantifications) can be downloaded from http://research.ncl.ac.uk/colonyzer/ChangSenescence/ (PDF, 52 KB)
- Table S1 - 60 genes tested by liquid senescence assay (PDF, 176 KB)
- Table S2 - Gene ontology analysis (processes) of RAD52-like genes from the solid screen in this study (PDF, 80 KB)
