# Supplementary material for: Genome-Wide Analysis to Identify Pathways Affecting Telomere-Initiated Senescence in Budding Yeast
Source: G3 (Bethesda). 2011 Aug 1;1(3):197–208. doi: 10.1534/g3.111.000216 (PMC3276134; doi:10.1534/g3.111.000216)
Supplement: Supporting Information [file supp_1.3.197_000216.pdf]

A

his3Δ<br>*est1Δ*	*his3Δ*<br>*est1Δ*	*his3Δ*<br>*est1Δ*	*his3Δ*<br>*est1Δ*	*his3Δ*<br>*est1Δ*	*his3Δ*<br>*est1Δ*	*his3Δ*<br>*est1Δ*	*his3Δ*<br>*est1Δ*	*his3Δ*<br>*est1Δ*	*his3Δ*<br>*est1Δ*	*his3Δ*<br>*est1Δ*	*his3Δ*<br>*est1Δ*
his3Δ<br>*est1Δ*	*est1Δ*<br>*est1Δ*	*rad57Δ*<br>*est1Δ*	*est3Δ*<br>*est1Δ*	*tel1Δ*<br>*est1Δ*	*rad55Δ*<br>*est1Δ*	*rad54Δ*<br>*est1Δ*	*asf1Δ*<br>*est1Δ*	*xrs2Δ*<br>*est1Δ*	*dcc1Δ*<br>*est1Δ*	*rif1Δ*<br>*est1Δ*	*his3Δ*<br>*est1Δ*
his3Δ<br>*est1Δ*	*rif2Δ*<br>*est1Δ*	*elg1Δ*<br>*est1Δ*	*pol32Δ*<br>*est1Δ*	*upf2Δ*<br>*est1Δ*	*upf3Δ*<br>*est1Δ*	*cdd73Δ*<br>*est1Δ*	*ydl118Δ*<br>*est1Δ*	*sum1Δ*<br>*est1Δ*	*rfm1Δ*<br>*est1Δ*	*rtf1Δ*<br>*est1Δ*	*his3Δ*<br>*est1Δ*
his3Δ<br>*est1Δ*	*hst3Δ*<br>*est1Δ*	*spt21Δ*<br>*est1Δ*	*hmo1Δ*<br>*est1Δ*	*mot3Δ*<br>*est1Δ*	*upf1Δ*<br>*est1Δ*	*kem1Δ*<br>*est1Δ*	*lea1Δ*<br>*est1Δ*	*mak10Δ*<br>*est1Δ*	*mak31Δ*<br>*est1Δ*	*mak3Δ*<br>*est1Δ*	*his3Δ*<br>*est1Δ*
his3Δ<br>*est1Δ*	*rrp8Δ*<br>*est1Δ*	*mrpl44Δ*<br>*est1Δ*	*csm3Δ*<br>*est1Δ*	*sla1Δ*<br>*est1Δ*	*rad27Δ*<br>*est1Δ*	*chk1Δ*<br>*est1Δ*	*ebs1Δ*<br>*est1Δ*	*rad9Δ*<br>*est1Δ*	*rad24Δ*<br>*est1Δ*	*rad52Δ*<br>*est1Δ*	*his3Δ*<br>*est1Δ*
his3Δ<br>*est1Δ*	*mre11Δ*<br>*est1Δ*	*exo1Δ*<br>*est1Δ*	*rad17Δ*<br>*est1Δ*	*hex3Δ*<br>*est1Δ*	*bnr1Δ*<br>*est1Δ*	*rho4Δ*<br>*est1Δ*	*ede1Δ*<br>*est1Δ*	*bmh1Δ*<br>*est1Δ*	*bmh2Δ*<br>*est1Δ*	*ddc1Δ*<br>*est1Δ*	*his3Δ*<br>*est1Δ*
his3Δ<br>*est1Δ*	*ctf18Δ*<br>*est1Δ*	*ctf8Δ*<br>*est1Δ*	*slx8Δ*<br>*est1Δ*	*tsa1Δ*<br>*est1Δ*	*sgs1Δ*<br>*est1Δ*	*rtt107Δ*<br>*est1Δ*	*rtt101Δ*<br>*est1Δ*	*mms1Δ*<br>*est1Δ*	*mms22Δ*<br>*est1Δ*	*rtt109Δ*<br>*est1Δ*	*his3Δ*<br>*est1Δ*
his3Δ<br>*est1Δ*	*his3Δ*<br>*est1Δ*	*his3Δ*<br>*est1Δ*	*his3Δ*<br>*est1Δ*	*his3Δ*<br>*est1Δ*	*his3Δ*<br>*est1Δ*	*his3Δ*<br>*est1Δ*	*his3Δ*<br>*est1Δ*	*his3Δ*<br>*est1Δ*	*his3Δ*<br>*est1Δ*	*his3Δ*<br>*est1Δ*	*his3Δ*<br>*est1Δ*

Red: Telomere length (Askree *et al.* 2004; Gatbonton *et al.* 2006);
Purple: DNA replication or repair (Collins *et al.* 2008);
Green: Genes of general interest.

B Passage1

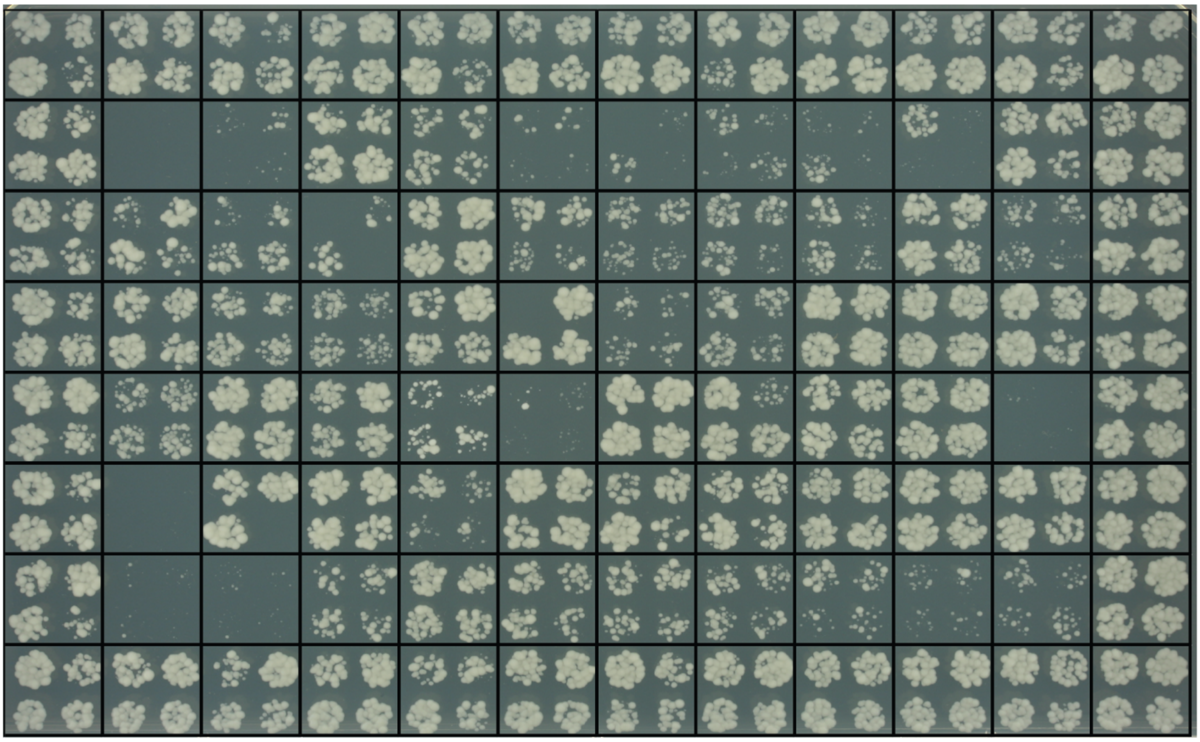

Figure S1 60 *yfgΔ est1Δ* strains arrayed in quadruplicate on a 384 format plate. (A) Strain map indicating the position of 60 genotypes on the 384 format plate in panel B; four replicates drawn in light grey. Red, purple, and green names indicate the reason for choosing particular genes for this study. (B) Photograph of a 384 format plate at passage 1 (48 hours after inoculation) from the liquid procedure (Figure 1A)

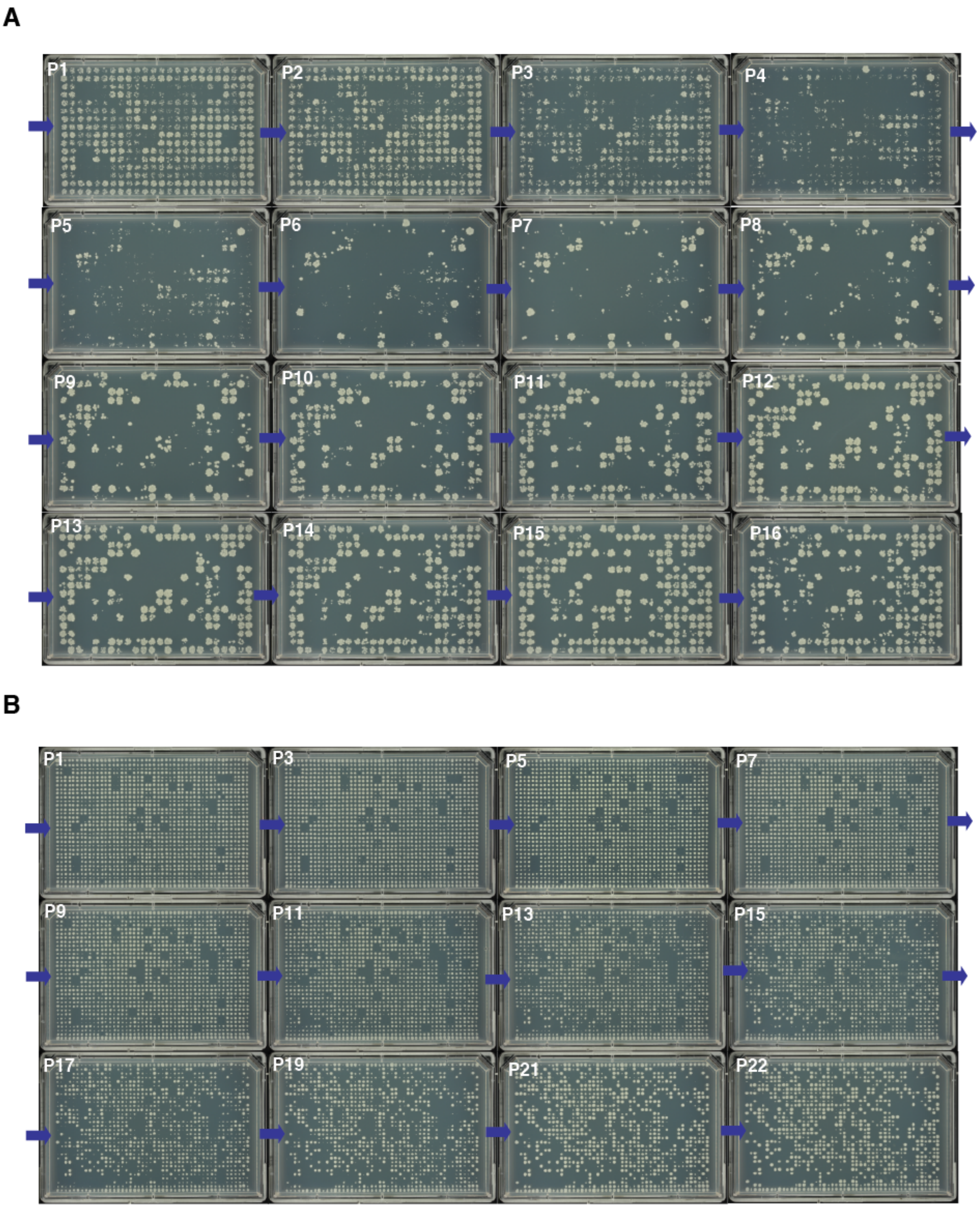

Figure S2 Example photographs from passaged cultures. (A) Photographs of 384 format agar plates for each of the 16 passages from the liquid procedure. (B) 12 sample photographs at various passages (indicated) for of one of the 1536 format plates from the solid procedure. Passage numbers are labelled on each photograph; P1-passage 1, etc.

A

	P1	P2	P3	P4	P5	P6	P7	P8	P9	P10	P11	P12	P13
**WT**	314	330	334	344	330	384	460	442	436	372	286	610	400
est3Δ	369	240	137	15	7	29	510	282	362	482	292	378	399
est1Δ	410	269	143	23	9	44	521	301	332	387	297	400	365
est3Δ est1Δ	212	120	85	19	76	110	310	230	305	386	255	388	303

B

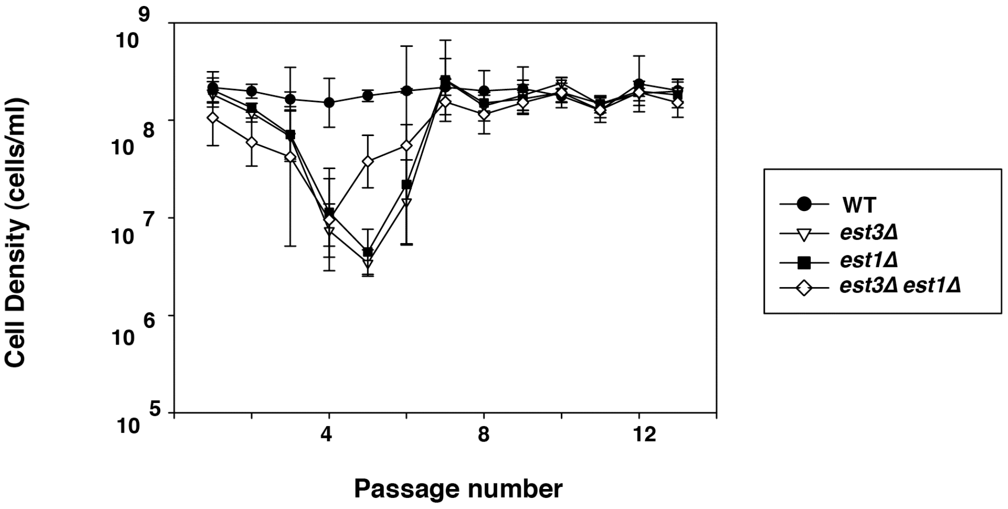

Figure S3 Low through-put senescence experiment in the W303 genetic background. (A) Six independent strains of each genotype in the W303 background were taken directly from germination plates and grown in liquid culture. Cell densities were counted every 23 hrs, followed by dilution to 5×10^5 cells/ml and continued incubation. Cell density reached by cells with the same genotype after 23 hours were recorded and averaged. The dilution factors at each passage were calculated and showed in the table, genotypes are indicated. (B) The data from (A) were plotted. Each symbol represents the average cell density reached by cells with the same genotype after 23 hours and the error bars represent the standard deviations.

A

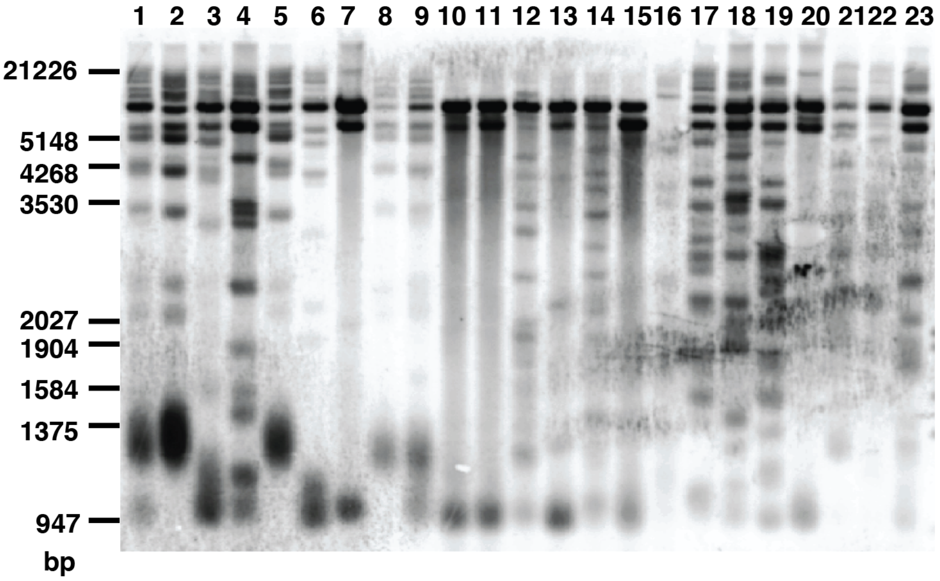

1. Wild type (DLY3799)
2. *sgs1Δ*
3. *sgs1Δ est1Δ* P1
4. *sgs1Δ est1Δ* P22
5. *rad17Δ*
6. *rad17Δ est1Δ* P1
7. *rad17Δ est1Δ* P22
8. *his3Δ*
9. *his3Δ est1Δ* P1
10. *his3Δ est1Δ* P22
11. *dcc1Δ est1Δ* P22
12. *cdc73Δ est1Δ* P22
13. *exo1Δ est1Δ* P22
14. *ebs1Δ est1Δ* P22
15. *upf3Δ est1Δ* P22
16. *rad27Δ est1Δ* P22
17. *ctf18Δ est1Δ* P22
18. *rif1Δ est1Δ* P22
19. *rad9Δ est1Δ* P22
20. *rif2Δ est1Δ* P22
21. *sae2Δ est1Δ* P22
22. *tel1Δ est1Δ* P22
23. *chk1Δ est1Δ* P22

P1-passage1
P22-passage22

Figure S4 Survivors were produced by passage 22 in the solid procedure. Genomic DNA prepared from the strains of different genotypes as indicated, digested with *XhoI* and subjected to Southern blot to detect telomeric Y' and "TG" fragments.

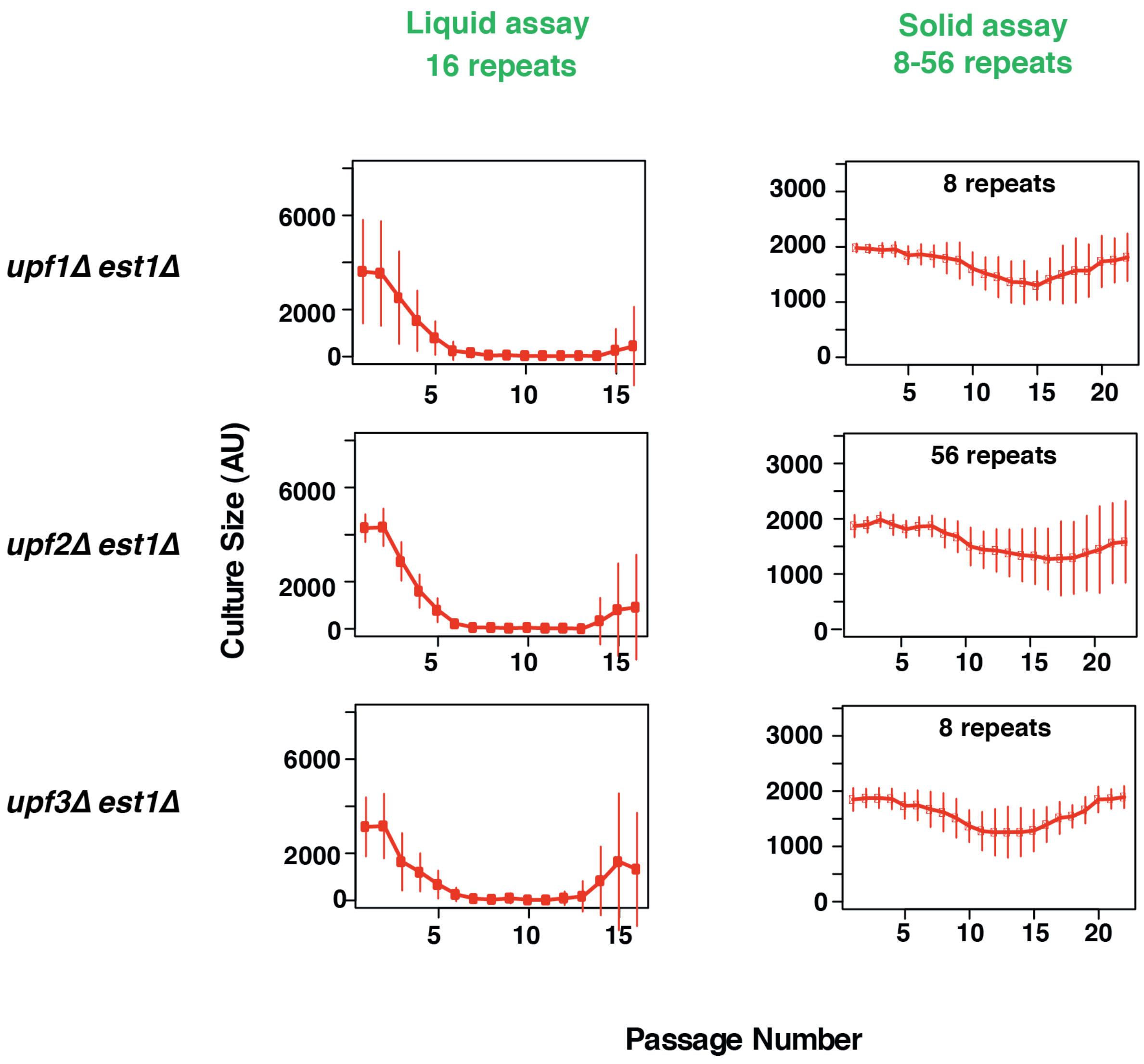

Figure S5 Nonsense mediated decay genes had consistent MDPs in both screens. *UPF1, UPF2,* and *UPF3* gene deletions had consistent MDPs patterns in both the liquid procedure and in the solid procedure. Replicate number and experiment type are indicated in green text.

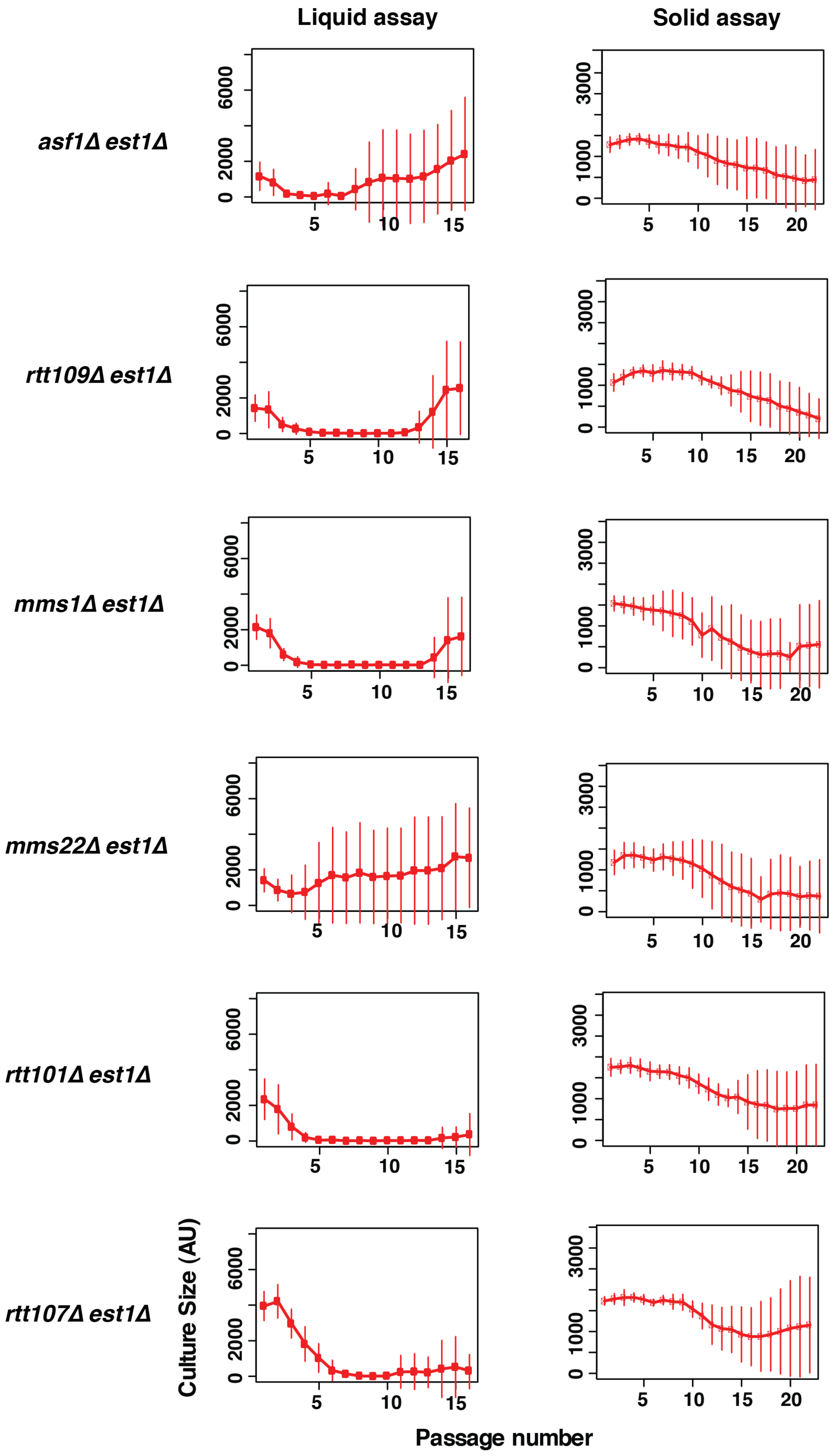

Figure S6 Genes affecting replication fork progression had an accelerated senescence phenotype when deleted in the *est1Δ* background. MDPs for *asf1Δ est1Δ*, *rtt109Δ est1Δ*, *mms1Δ est1Δ*, *mms22Δ est1Δ*, *rtt101Δ est1Δ*, and *rtt107Δ est1Δ* indicating a fast senescence phenotype in both the liquid and solid assay.

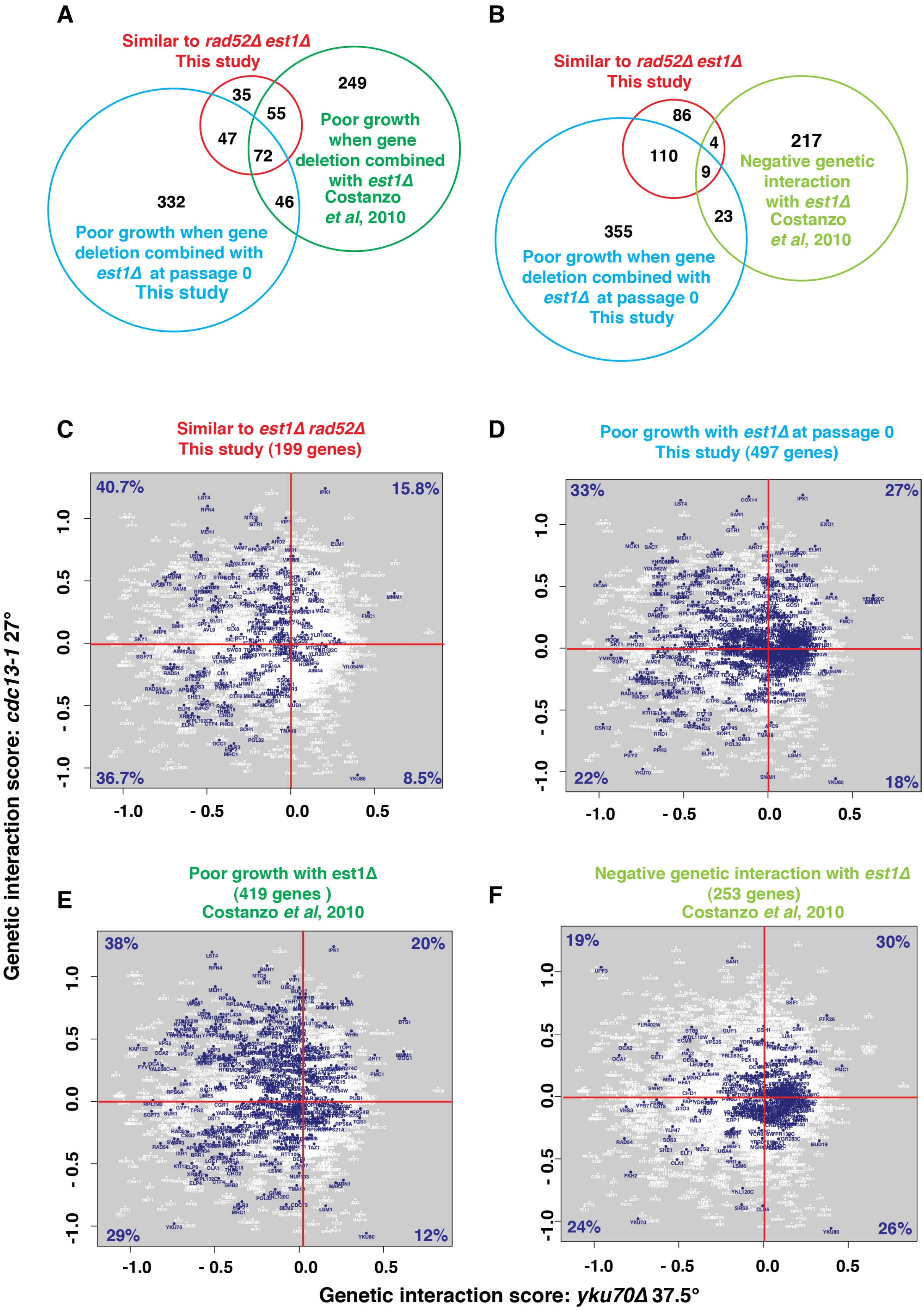

Figure S7 Repeated passage of cultures provides useful information about telomere-dependent senescence. (A) Venn diagram demonstrating the overlap between poor growers from our initial SGA (blue circle), poor growers from the *est1Δ* SGA by (Costanzo *et al.* 2010b) (green circle) and our accelerated senescence class defined by repeated passaging, similar to *rad52Δ*, Figure 4B, Supporting File S7 (red circle). Poor growers are defined here as deletions whose fitness is more than one standard deviation less than the mean fitness across all deletions in the library. (B) As for panel A,

except the green circle in this panel represents genes classified as having a lenient negative interaction with *est1Δ* by Costanzo et al. (C) *yku70Δ* vs *cdc13-1* genetic interaction profile (see Figure 6) with our accelerated senescence class overlaid (blue). (D) *yku70Δ* vs *cdc13-1* genetic interaction profile with poor growers from our initial SGA overlaid in blue (E) *yku70Δ* vs *cdc13-1* genetic interaction profile with poor growers from the *est1Δ* SGA by Costanzo et al. overlaid (blue) (F) *yku70Δ* vs *cdc13-1* genetic interaction profile with genes classified as having a lenient negative interaction with *est1Δ* by Costanzo et al. overlaid (blue, see Figure 4A, Table ST4).

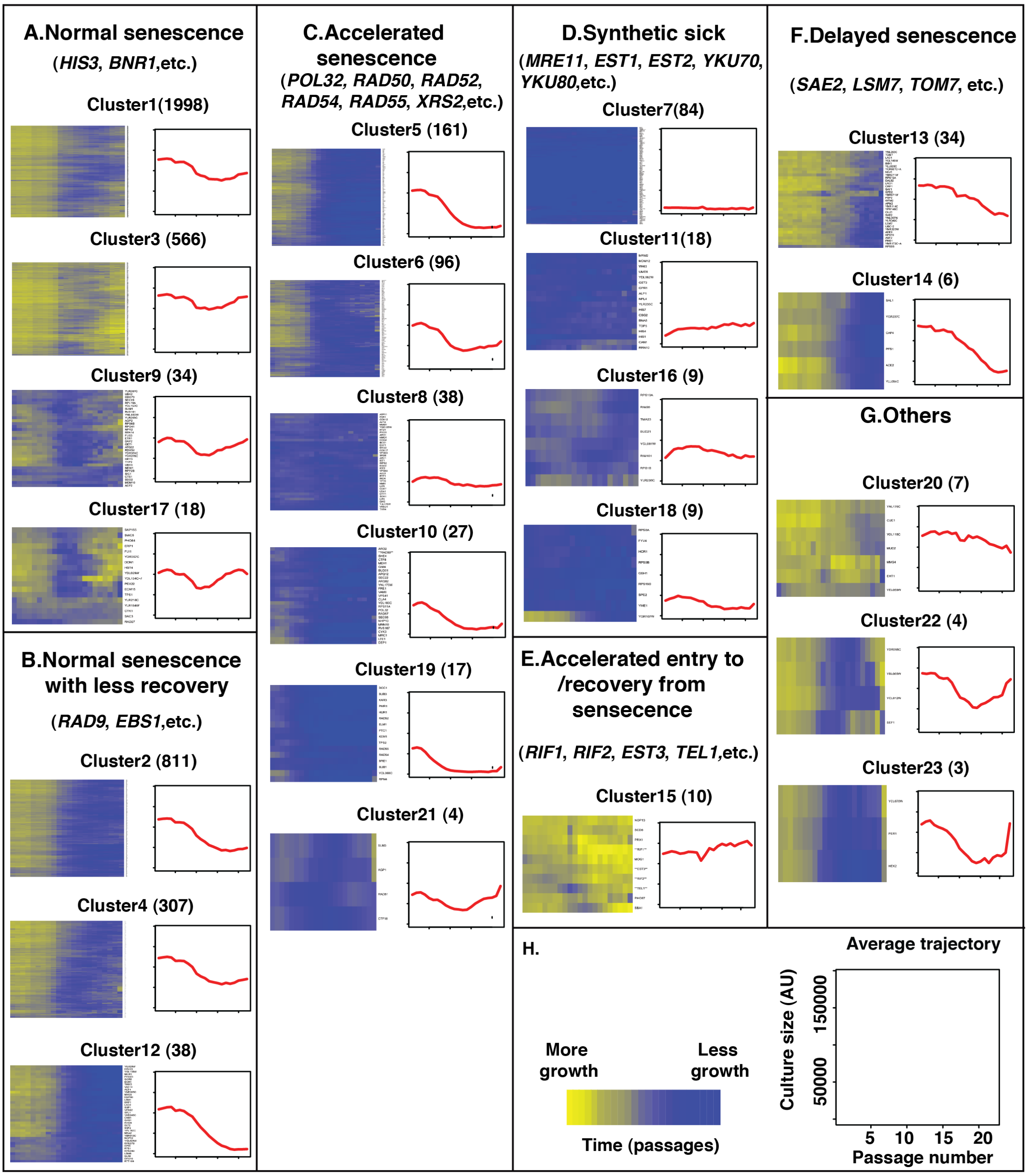

Figure S8 Unsupervised hierarchical QT clustering identifies 23 different MDP classes. Heat map representation of cluster MDPs on left, average MDPs on right. 23 automatically generated classes were combined manually into 8 functionally meaningful classifications (A-H).

Files S1-S13
All supporting files listed below (together with raw images and initial culture size quantifications) can be downloaded from our Supplemental Data website: http://research.ncl.ac.uk/colonyzer/ChangSenescence/

File S1. Liquid screen MDP plots.
Mean Density Profiles for each of 60 strains grown in the liquid culture screen. Red points are average culture size for all available repeats at passages 1 - 16. Vertical red lines show standard deviation for all repeats.
File S2. Solid screen MDP plots.
Mean Density Profiles for each of ~4300 strains grown in the solid agar screen. Red points are average culture size for all available repeats at passages 1 - 22. Vertical red lines show standard deviation for all repeats.
File S3. Manual classification of MDPs from liquid screen.
Manual functional clustering of the 60 gene deletions investigated in the liquid culture screen. MDPs for each cluster member plotted in panels A – P. 3 main categories are indicated in black text; functionally related sub-categories are defined by panels and titled in green text: (A) Recombination repair (B) *CTF18* RFC-like complex (C) Nonsense mediated decay. (D) Histone acetyltransferase. (E) MRX complex. (F) *SLX5-SLX8* STUbL Complex. (G) Ubiquitin-conjuating enzyme. (H) AF1 complex. (I) *SUM1/RFM1* repressor complex. (J) Mean plots of gene deletions that accelerate senescence. (K) Mean plots of gene deletions that accelerated entry to and escaped from senescence. (L) Subunit of N-terminal acetyltrasferase. (M) DNA damage checkpoint. (N) 14-3-3 proteins. (O&P) Gene deletions that do not affect senescence. Dashed lines indicate genes that are not classified in any particular group.
File S4. MDPs for all 4299 strains in the solid screen.
Tab-delimited text file containing quantitative MDPs (culture size profiles with increasing passage). Culture sizes are in arbitrary units. Note that photo 1 corresponds to the first photograph taken after the SGA. Passage 1 in the manuscript corresponds to photo 2.
File S5. MDPs similar to that of the *his3Δ est1Δ* archetype from the solid screen.
Tab-delimited text file listing Pearson's correlation coefficient (c) between the MDP of the archetype strain *his3Δ est1Δ* and all other deletions in the library. Gene deletions are ranked by correlation coefficient and thereby profile similarity. Root Mean Square difference (RMS) between the MDP of each strain and that of the archetype is also reported, together with an estimate of the area under the MDP of each strain (Integral).
File S6. MDPs similar to that of the *rad52Δ est1Δ* archetype from the solid screen.
As for File S5.
File S7. MDPs similar to that of the *rad52Δ est1Δ* archetype from the solid screen which are also poor growers.
As for S5, except similarity is defined as deletions in the library with Corr > 0.5 and Culture Size at passage 2 < 1680 (see Figure 4).
File S8. MDPs similar to that of the *rif1Δ est1Δ* archetype from the solid screen.
As for File S5.
File S9. Gene lists used in the Venn diagram in Figure S7
Tab-delimited lists of gene names classified as having similar MDPs to the *rad52Δ est1Δ* archetype (RAD52Archetype), classified as being poor growers after the SGA presented in this work (SGASick), classified as being poor growers in the *est1Δ* double mutant fitness data set Costanzo et al. 2010 (Supporting data file S1) (CostanzoSick) or classified as having a (lenient) negative interaction with *est1Δ* by Costanzo et al. 2010 (CostanzoNegGIS). Poor growers were defined as having a fitness or culture size less than the mean by more than one standard deviation.
File S10. Intersection classes in Venn diagram Figure S7.
Venn diagram from Figure S7 repeated with letters classifying groups of gene deletions from overlapping gene lists as defined in File S9.
File S11. Gene members of interaction classes in Venn diagram in File S10.
Tab-delimited member lists for gene deletion categories defined in File S10.
File S12. 23 clusters from unsupervised QT clustering of MDPs from solid screen.
Clusters are represented by MDP heat maps showing culture size variation with passage (V1-V22), dendograms linking MDPs (left) the names of gene deletions for each MDP (right) together with alternating plots of the average MDP for all members of each cluster (red curve, passage number on x-axis, culture size on y-axis).
File S13. Gene members of clusters presented in File S12
Tab-delimited text file classifying each ORF in the deletion library by cluster membership.

Table S1 60 genes tested by liquid senescence assay

Gene	*Senescence phenotype	**Telomere length screens	Gene ontology annotation	***References
EST3	AERS	VS	telomerase component	*est2Δ est3Δ/est1Δ est3Δ/tlc1Δ est3Δ* showed normal senescence (Lendvay *et al.* 1996)
RIF1	AERS	VL	telomere binding protein	*rif1Δ est2Δ* showed normal senescence (Anbalagan S *et al.* 2011); *rif1Δ est2Δ* accelerated entry to /recovery from senescence (Chang *et al.* 2011)
RIF2	AERS	VL	telomere binding protein	*rif2Δ est2Δ* accelerated entry to/recovery from senescence (Chang *et al.* 2011)
EST1	AS	VS	telomerase component	
MRE11	AS	VS	DNA repair/ MRX complex	Normal (Le *et al.* 1999)
XRS2	AS	VS	DNA repair/MRX complex	Normal (Le *et al.* 1999)
RAD52	AS		recombinational repair of double-strand breaks in DNA	*rad52Δ est1Δ and rad52Δ tlc1Δ* accelerated senescence (Abdallah *et al.* 2010; Le *et al.* 1999; LeBel *et al.* 2009; Lee *et al.* 2007; Lundblad and Blackburn 1993)
RAD54	AS		recombinational repair of double-strand breaks in DNA	*rad54Δ tlc1Δ* has severe growth defect (Le *et al.* 1999)
RAD55	AS		recombinational repair of double-strand breaks in DNA	
RAD57	AS		recombinational repair of double-strand breaks in DNA	*rad57Δ tlc1Δ* has severe growth defect (Le *et al.* 1999)
UPF1 (NAM7)	AS	VS	nonsense mediated deccay	*upf1Δ* with *tlc1Δ/est1Δ/est2Δ/est3Δ* delayed senescence measured by a different method (Enomoto *et al.* 2004)
UPF2 (NMD2)	AS	S	nonsense mediated deccay	*upf2Δ* with *tlc1Δ/est1Δ/est2Δ/est3Δ* delayed senescence measured by a different method (Enomoto *et al.* 2004)
UPF3	AS	S	nonsense mediated deccay	*upf3Δ* with *tlc1Δ/est1Δ/est2Δ/est3Δ* delayed senescence measured by a different method (Enomoto *et al.* 2004)

DCC1	AS	ss	Sister chromatid cohesion	
CTF18	AS	Literature reported	Sister chromatid cohesion	
CTF8	AS	ss	Sister chromatid cohesion	
ELG1	AS	L	RFC complex	
RTT101	AS		Histone acetyltransferase/involved in NHEJ	
RTT109	AS		Histone acetyltransferase/involved in NHEJ	
RTT107	AS		Mms22-dependent DNA repair during S phase/interacts with Mms22p and Slx4p	
MMS1	AS		Subunit of an E3 ubiquitin ligase complex/resolving replication intermediates	*mms1Δ tlc1Δ* accelerated senescence and failed to recover (ABDALLAH *et al.* 2010)
MMS22	AS		Ubiquitin-conjugating enzyme variant involved in error-free post replication repair	
CDC73	AS	S	PAF1 complex	
RTF1	AS	ss	PAF1 complex	
SLX8	AS	sl,	Subunit of the Slx5-Slx8 SUMO-targeted ubiquitin ligase (STUbL) complex	*slx8Δ tlc1Δ* accelerated senescence (AZAM *et al.* 2006)
SLX5 (HEX3)	AS		Subunit of the Slx5-Slx8 SUMO-targeted ubiquitin ligase (STUbL) complex	*slx5Δ tlc1Δ* accelerated senescence (AZAM *et al.* 2006)

RRP8	AS	L	pre-rRNA processing/methyltransferase	
SPT21	AS	ss	regulator of histone gene transcription	
POL32	AS	sl	Polymerase delta subunit	*pol32Δ tlc1Δ* accelerated senescence (Lydeard *et al.* 2007)
SGS1	AS		Nucleolar DNA helicase of the RecQ family	*sgs1Δ tlc1Δ* accelerated senescence (Azam *et al.* 2006; Lee *et al.* 2007)
RAD27	AS	VL	flap-endonuclease	*rad27Δ* with *est1Δ/tlc1Δ/est3Δ/cdc13-2* accelerated senescence (Parenteau and Wellinger 2002)
HMO1	AS	L	HMG-box protein	
TEL1	AS	VS	PIK homologue	*tel1Δ tlc1Δ showed* normal senescence (Enomoto *et al.* 2002) ; *tel1Δ tlc1Δ* delayed senescence (Ritchie *et al.* 1999), *tel1Δ tlc1Δ mec1Δ sml1Δ* is normal (Chan *et al.* 2001); *tel1Δ tlc1Δ* delayed senescence (Abdallah *et al.* 2010)
KEM1	AS	S	RNA degradation	
YDL118	AS	ss	Unknown	
LEA1	AS	L	RNA splicing	
SUM1	AS	S	SUM1/RFM1 repressor complex	
RFM1	AS	S	SUM1/RFM1 repressor complex	
TSA1	AS		Thioredoxin peroxidase/ribosome-associated and free cytoplasmic antioxidant	
ASF1	AS		Nucleosome assembly factor/chromatin assembly and disassembly	
SLA1	AS		Cytoskeletal binding protein	

RHO4	AS		Non-essential small GTPase of the Rho/Rac subfamily of Ras-like proteins	
EDE1	AS		Key endocytic protein	
EXO1	Normal	Literature reported	5'-3' exonuclease and flap-endonuclease	*exo1Δ tlc1Δ* delayed senescence (Maringele and Lydall 2004)
EBS1	Normal	ss	nonsense mediated decay	
RAD9	Normal	Literature reported	DNA damage checkpoint effector	*rad9Δ tlc1Δ* had less G2/M arrested cells (Ijpma and Greider 2003)
RAD17	Normal	Literature reported	DNA damage checkpoint effector	
DDC1	Normal	Literature reported	DNA damage checkpoint effector	
RAD24	Normal	Literature reported	DNA damage checkpoint effector	*rad24Δ tlc1Δ* had less G2/M arrested cells (Ijpma and Greider 2003)
CHK1	Normal		DNA damage checkpoint effector	
BMH1	Normal	Literature reported	14-3-3 protein	
BMH2	Normal	Literature reported	14-3-3 protein	
MRPL44	Normal	ss	mitochondrial ribosomal protein	
MOT3	Normal	ss	POLII transcription	
HST3	Normal		Member of the Sir2 family of NAD(+)-dependent protein deacetylases	
CSM3	Normal		Replication fork associated factor	
BNR1	Normal		Formin	
HIS3	Normal		Imidazoleglycerol-phosphate dehydratase	Control strain
MAK3	Normal	L	N-terminal acetyltransferase complex	
MAK10	Normal	L	N-terminal acetyltransferase complex	
MAK31	Normal	L	N-terminal acetyltransferase complex	

Legend: Examining of the 60 genes selected for the liquid procedure in this study. The references quoted were indicated in the table and included below.

*Senescence phenotype abbreviations: AS- accelerated senescence, AERS-accelerated entry to and recovery from senescence.
Normal: normal entry to and recovery from senescence.
** Telomere phenotype abbreviations (Askree *et al.* 2004; Gatbonton *et al.* 2006; Shachar *et al.* 2008): VS - very short, S - short, ss - slightly short, sl - slightly long, L - long, VL - very long.
*** Conflicts in data may be due to differences in ways senescence was measured.

References

Abdallah, P., P. Luciano, K. W. Runge, M. Lisby, V. Geli *et al.*, 2010 A two-step model for senescence triggered by a single critically short telomere (vol 11, pg 988, 2009). Nature Cell Biology **12:** 520-520.

Anbalagan S, Bonetti D, Lucchini G and L. MP., 2011 Rif1 Supports the Function of the CST Complex in Yeast Telomere Capping. PLos Genetics **7**.

Askree, S. H., T. Yehuda, S. Smolikov, R. Gurevich, J. Hawk *et al.*, 2004 A genome-wide screen for Saccharomyces cerevisiae deletion mutants that affect telomere length. Proceedings of the National Academy of Sciences of the United States of America **101:** 8658-8663.

Azam, M., J. Y. Lee, V. Abraham, R. Chanoux, K. A. Schoenly *et al.*, 2006 Evidence that the S.cerevisiae Sgs1 protein facilitates recombinational repair of telomeres during senescence. Nucleic Acids Research **34:** 506-516.

Chan, S. W. L., J. Chang, J. Prescott and E. H. Blackburn, 2001 Altering telomere structure allows telomerase to act in yeast lacking ATM kinases. Current Biology **11:** 1240-1250.

Chang, M., J. C. Dittmar and R. Rothstein, 2011 Long telomeres are preferentially extended during recombination-mediated telomere maintenance. Nature structural and molecular biology.

Enomoto, S., L. Glowczewski and J. Berman, 2002 MEC3, MEC1, and DDC2 are essential components of a telomere checkpoint pathway required for cell cycle arrest during senescence in Saccharomyces cerevisiae. Molecular Biology of the Cell **13:** 2626-2638.

Enomoto, S., L. Glowczewski, J. Lew-Smith and J. G. Berman, 2004 Telomere cap components influence the rate of senescence in telomerase-deficient yeast cells. Molecular and Cellular Biology **24:** 837-845.

Gatbonton, T., M. Imbesi, M. Nelson, J. M. Akey, D. M. Ruderfer *et al.*, 2006 Telomere length as a quantitative trait: Genome-wide survey and genetic mapping of telomere length-control genes in yeast. Plos Genetics **2:** 304-315.

Ijpma, A. S., and C. W. Greider, 2003 Short telomeres induce a DNA damage response in Saccharomyces cerevisiae. Molecular Biology of the Cell **14:** 987-1001.

Le, S., J. K. Moore, J. E. Haber and C. W. Greider, 1999 RAD50 and RAD51 define two pathways that collaborate to maintain telomeres in the absence of telomerase. Genetics **152:** 143-152.

LeBel, C., E. Rosonina, D. C. F. Sealey, F. Pryde, D. Lydall *et al.*, 2009 Telomere Maintenance and Survival in Saccharomyces cerevisiae in the Absence of Telomerase and RAD52. Genetics **182:** 671-684.

Lee, J. Y., M. Kozak, J. D. Martin, E. Pennock and F. B. Johnson, 2007 Evidence that a RecQ helicase slows senescence by resolving recombining telomeres. Plos Biology **5:** 1334-1344.

Lendvay, T. S., D. K. Morris, J. Sah, B. Balasubramanian and V. Lundblad, 1996 Senescence mutants of Saccharomyces cerevisiae with a defect in telomere replication identify three additional EST genes. Genetics **144:** 1399-1412.

Lundblad, V., and E. H. Blackburn, 1993 AN ALTERNATIVE PATHWAY FOR YEAST TELOMERE MAINTENANCE RESCUES EST1-SENESCENCE. Cell **73:** 347-360.

Lydeard, J. R., S. Jain, M. Yamaguchi and J. E. Haber, 2007 Break-induced replication and telomerase-independent telomere maintenance require Pol32. Nature **448:** 820-U810.

Maringele, L., and D. Lydall, 2004 EXO1 plays a role in generating type I and type II survivors in budding yeast. Genetics **166:** 1641-1649.

Parenteau, J., and R. J. Wellinger, 2002 Differential processing of leading- and lagging-strand ends at Saccharomyces cerevisiae telomeres revealed by the absence of Rad27p nuclease. Genetics **162:** 1583-1594.

Ritchie, K. B., J. C. Mallory and T. D. Petes, 1999 Interactions of TLC1 (which encodes the RNA subunit of telomerase), TEL1, and MEC1 in regulating telomere length in the yeast Saccharomyces cerevisiae. Molecular and Cellular Biology **19:** 6065-6075.

Shachar, R., L. Ungar, M. Kupiec, E. Ruppin and R. Sharan, 2008 A systems-level approach to mapping the telomere length maintenance gene circuitry. Molecular Systems Biology **4**.

Table S2 Gene ontology analysis (processes) of RAD52-like genes from the solid screen in this study

GO-Slim term	Genes annotated to the term
Transport	*AGP1, APQ12, ATG9, AVL9, AVT5, CCZ1, CIK1, CLA4, DRS2, FKS1, GCS1, GET2, GOS1, GTR1, KAR3, LST4, MMM1, NUM1, PEX12, PEX14, PEX19, PEX5, PMR1, RIM8, RPS18B, SEC28, SEC66, SGF73, SKY1, SLG1, SNX4, SPC2, SPF1, SSO2, STE20, THR4, TIM18, TLG2, UBP3, UBX2, VAM3, VAM6, VAM7, VPS1, VPS4, VPS41, VPS5, VPS51, VTH1, YDL183C, YPT7, ZRC1*
Response to stress	*ASF1, ATG9, BRE1, CAC2, CCZ1, CKB1, CTF18, CTF4, CTF8, DCC1, ELM1, GSH1, HDA1, MGA2, MMS1, MMS22, MRC1, MRE11, NHP10, NPL4, OPI1, PHO5, PHO80, POL32, RAD50, RAD52, RAD54, RAD55, RAD57, RMI1, RPN4, RTT109, SIC1, SIN3, SLG1, SLX5, SLX8, SNF6, SOH1, STB5, STE20, TAX4, TIM18, TMA19, TPS2, UBI4, URM1, VAM3, VPS75, XRS2, YKU80*
RNA metabolic process	*ASF1, BRE1, CAC2, CKB1, DEG1, ELP2, ELP3, ELP4, ELP6, GRS1, GTR1, HAP4, HDA1, HTZ1, INO2, IPK1, MED1, MGA2, MMS1, MRC1, MRE11, NCS6, NOP12, OPI1, PHO80, POL32, RPL35B, RPN4, RPS11B, RPS16A, RPS18B, RTF1, RTT109, SDS3, SGF11, SGF73, SIN3, SKY1, SNF4, SNF6, SOH1, STB5, SWD1, SWD3, URM1, YGR122W, YKU80, YPL205C, ZDS2*
Chromosome organization	*ARP6, ASF1, BRE1, CAC2, CIK1, CTF18, CTF4, CTF8, DCC1, HDA1, KAR3, LGE1, MMS22, MRC1, NHP10, RAD50, RAD51, RAD52, RAD54, RAD57, RMI1, RTF1, RTS1, RTT109, SDS3, SGF11, SGF29, SGF73, SIN3, SLX5, SLX8, SNF6, SOH1, SWD1, SWD3, VPS75, XRS2, YKU80*
Transcription, DNA-dependent	*ASF1, BRE1, CAC2, CKB1, ELP2, ELP3, ELP4, ELP6, GRS1, GTR1, HAP4, HDA1, HTZ1, INO2, MED1, MGA2, MRC1, MRE11, OPI1, PHO80, RPN4, RTF1, RTT109, SDS3, SGF11, SGF73, SIN3, SNF4, SNF6, SOH1, STB5, SWD1, SWD3, YGR122W, YKU80, YPL205C, ZDS2*
Protein modification process	*ASF1, BRE1, CKB1, CLA4, DIA2, ELM1, ELP2, ELP6, HDA1, HPM1, LGE1, MUB1, NCS6, OST4, PHO80, RTF1, RTS1, RTT109, SDS3, SGF11, SGF29, SGF73, SIC1, SIN3, SKY1, SLX5, SLX8, SNF4, STE20, SWD1, SWD3, UBI4, UBP3, UBP6, URM1, VPS75*
DNA metabolic process	*BRE1, CAC2, CTF18, CTF4, CTF8, DCC1, DIA2, HUR1, LGE1, MMS1, MMS22, MRC1, MRE11, NHP10, POL32, RAD50, RAD51, RAD52, RAD54, RAD55, RAD57, RIM1, RPN4, RTT109, SIN3, SLX5, SLX8, SNF6, SOH1, SWD1, SWD3, VPS75, XRS2, YKU80*
Cell cycle	*BRE1, CIK1, CLA4, CTF18, CTF4, CTF8, DCC1, ELM1, FAR1, GCS1, KAR3, MMS22, MRC1, MRE11, PHO80, RAD50, RAD51, RAD52, RAD55, RAD57, RIM8, RMI1, RPN4, RTS1, SAP155, SHE1, SIC1, SIN3, SMI1, SOH1, STE20, XRS2, ZDS2*
Vesicle-mediated transport	*AVL9, CCZ1, DRS2, FKS1, GCS1, GET2, GOS1, LST4, PMR1, RIM8, SEC28, SLG1, SNX4, SSO2, THR4, TLG2, UBP3, VAM3, VAM6, VAM7, VPS1, VPS4, VPS41, VPS5, VPS51, VTH1, YPT7*
Cellular membrane organization	*APQ12, ATG9, CCZ1, DRS2, FKS1, GET2, GOS1, MEH1, MMM1, RIM8, SEC28, SLG1, SNX4, SSO2, THR4, TIM18, TLG2, VAM10, VAM3, VAM6, VAM7, VPS1, VPS4, VPS41, YPT7*
Biological process unknown	*AIM4, AIM44, IES2, IES5, MTC1, MTC5, PAR32, RTC2, YBR174C, YDR149C, YGL024W, YGL081W, YGR125W, YGR182C, YIL054W, YLR065C, YLR108C, YLR287C, YML012C-A, YNL170W, YPL102C*
Meiosis	*BRE1, CIK1, KAR3, MMS22, MRE11, RAD50, RAD51, RAD52, RAD55, RAD57, RIM8, RTS1, SOH1, XRS2*

Response to chemical stimulus	*CLA4, ELM1, FAR1, GSH1, NPL4, OPI1, RPN4, SKY1, STB5, STE20, TIM18, TMA19, URM1*
Protein complex biogenesis	*ATG9, ATP11, COX12, COX23, FMC1, GIM4, PEX14, PEX5, PKR1, SGF73, VAM6, VPS4, VPS41*
Cellular amino acid metabolic process	*ARO1, ARO2, GRS1, GSH1, GSH2, HOM6, PDC5, SPE1, SPE2, SPE3, THR4, ZRC1*
Cellular lipid metabolic process	*APQ12, CHO2, ETR1, INO2, INP52, MGA2, OAR1, OPI1, OPI3, PSD1, SCT1, TLG2*
Peroxisome organization	*ATG9, CCZ1, PEX12, PEX14, PEX19, PEX5, SLG1, SNF4, SNX4, TLG2, VPS1, VPS51*
Chromosome segregation	*CIK1, CTF18, CTF4, CTF8, DCC1, KAR3, MMS22, MRC1, RMI1, RTS1, SOH1*
Mitochondrion organization	*ATP11, COX12, COX23, FMC1, GEM1, GRS1, MMM1, MRPL36, NUM1, RIM1, TIM18*
Translation	*EAP1, GRS1, MRPL36, RPL21B, RPL35B, RPS11B, RPS16A, RPS18B, RPS4A, TEF4, TMA19*
Vesicle organization	*GOS1, SEC28, SSO2, TLG2, VAM3, VAM6, VAM7, VPS4, VPS41, VPS51*
Vacuole organization	*ATG9, CLA4, STE20, VAM10, VAM3, VAM6, VAM7, VPS41, YPT7*
Cellular carbohydrate metabolic process	*ELM1, FKS1, MAL12, OST4, PDC5, PFK2, SNF4, SOL4, TPS2*
Signaling	*CLA4, FAR1, MRC1, OPI1, PHO80, SLG1, STE20, TAX4*
Sporulation resulting in formation of a cellular spore	*MRE11, RIM21, RIM9, SSO2, UBI4, XRS2, YPL205C*
Cytoskeleton organization	*CIK1, GCS1, NUM1, SHE1, SLG1, VPS1*
Ribosome biogenesis	*DRS2, NOP12, RPL35B, RPS11B, RPS16A, RPS18B*
Cytokinesis	*CLA4, CTS1, CYK3, ELM1, STE20*
Generation of precursor metabolites and energy	*CYT1, ETR1, OAR1, PDC5, PFK2*
Cofactor metabolic process	*CAT5, SOL4, SPE1, SPE2, SPE3*
Cell budding	*CLA4, ELM1, MUB1, URM1, VPS51*
Cellular homeostasis	*MEH1, PHO80, SKY1, SPF1, ZRC1*
Conjugation	*CIK1, FAR1, KAR3, STE20*
Pseudohyphal growth	*DFG16, ELM1, SOK2, STE20*
Fungal-type cell wall organization	*ECM8, KRE1, SLG1, TAX4*
Heterocycle metabolic process	*AAH1, IMD3, PDC5, SOL4*
Cellular aromatic compound metabolic process	*AAH1, ARO1, ARO2, PDC5*
Cellular protein catabolic process	*DIA2, NPL4, UBX2, VMS1*
Cellular respiration	*CYT1, ETR1, OAR1*
Nucleus organization	*APQ12, CIK1, KAR3*
Vitamin metabolic process	*SPE1, SPE2, SPE3*
Cellular component morphogenesis	*ELM1, SSO2*
Transposition	*MMS1, RTT109*
Protein folding	*EGD2*

Table legend: *RAD52*-like genes in this study were analysed using SGD gene ontology slim mapper (http://www.yeastgenome.org/cgi-bin/GO/goSlimMapper.pl). “Yeast GO-Slim: Process” GO set was used to determine the list of genes that involved in different biological processes.
