# Supplementary material for: Genome-Wide Analysis to Identify Pathways Affecting Telomere-Initiated Senescence in Budding Yeast
Source: G3 (Bethesda). 2011 Aug 1;1(3):197–208. doi: 10.1534/g3.111.000216 (PMC3276134; doi:10.1534/g3.111.000216)
Supplement: Supporting Information [file supp_1.3.197_FigureS1.pdf]

A

his3Δ est1Δ	*his3Δ est1Δ*	*his3Δ est1Δ*	*his3Δ est1Δ*	*his3Δ est1Δ*	*his3Δ est1Δ*	*his3Δ est1Δ*	*his3Δ est1Δ*	*his3Δ est1Δ*	*his3Δ est1Δ*	*his3Δ est1Δ*	*his3Δ est1Δ*
his3Δ est1Δ	*est1Δ est1Δ*	*rad57Δ est1Δ*	*est3Δ est1Δ*	*tel1Δ est1Δ*	*rad55Δ est1Δ*	*rad54Δ est1Δ*	*asf1Δ est1Δ*	*xrs2Δ est1Δ*	*dcc1Δ est1Δ*	*rif1Δ est1Δ*	*his3Δ est1Δ*
his3Δ est1Δ	*rif2Δ est1Δ*	*elg1Δ est1Δ*	*pol32Δ est1Δ*	*upf2Δ est1Δ*	*upf3Δ est1Δ*	*cdd73Δ est1Δ*	*ydl118Δ est1Δ*	*sum1Δ est1Δ*	*rfm1Δ est1Δ*	*rtf1Δ est1Δ*	*his3Δ est1Δ*
his3Δ est1Δ	*hst3Δ est1Δ*	*spt21Δ est1Δ*	*hmo1Δ est1Δ*	*mot3Δ est1Δ*	*upf1Δ est1Δ*	*kem1Δ est1Δ*	*lea1Δ est1Δ*	*mak10Δ est1Δ*	*mak31Δ est1Δ*	*mak3Δ est1Δ*	*his3Δ est1Δ*
his3Δ est1Δ	*rrp8Δ est1Δ*	*mrpl44Δ est1Δ*	*csm3Δ est1Δ*	*sla1Δ est1Δ*	*rad27Δ est1Δ*	*chk1Δ est1Δ*	*ebs1Δ est1Δ*	*rad9Δ est1Δ*	*rad24Δ est1Δ*	*rad52Δ est1Δ*	*his3Δ est1Δ*
his3Δ est1Δ	*mre11Δ est1Δ*	*exo1Δ est1Δ*	*rad17Δ est1Δ*	*hex3Δ est1Δ*	*bnr1Δ est1Δ*	*rho4Δ est1Δ*	*ede1Δ est1Δ*	*bmh1Δ est1Δ*	*bmh2Δ est1Δ*	*ddc1Δ est1Δ*	*his3Δ est1Δ*
his3Δ est1Δ	*ctf18Δ est1Δ*	*ctf8Δ est1Δ*	*slx8Δ est1Δ*	*tsa1Δ est1Δ*	*sgs1Δ est1Δ*	*rtt107Δ est1Δ*	*rtt101Δ est1Δ*	*mms1Δ est1Δ*	*mms22Δ est1Δ*	*rtt109Δ est1Δ*	*his3Δ est1Δ*
his3Δ est1Δ	*his3Δ est1Δ*	*his3Δ est1Δ*	*his3Δ est1Δ*	*his3Δ est1Δ*	*his3Δ est1Δ*	*his3Δ est1Δ*	*his3Δ est1Δ*	*his3Δ est1Δ*	*his3Δ est1Δ*	*his3Δ est1Δ*	*his3Δ est1Δ*

Red: Telomere length (Askree *et al.* 2004; Gatbonton *et al.* 2006);
Purple: DNA replication or repair (Collins *et al.* 2008);
Green: Genes of general interest.

B Passage1

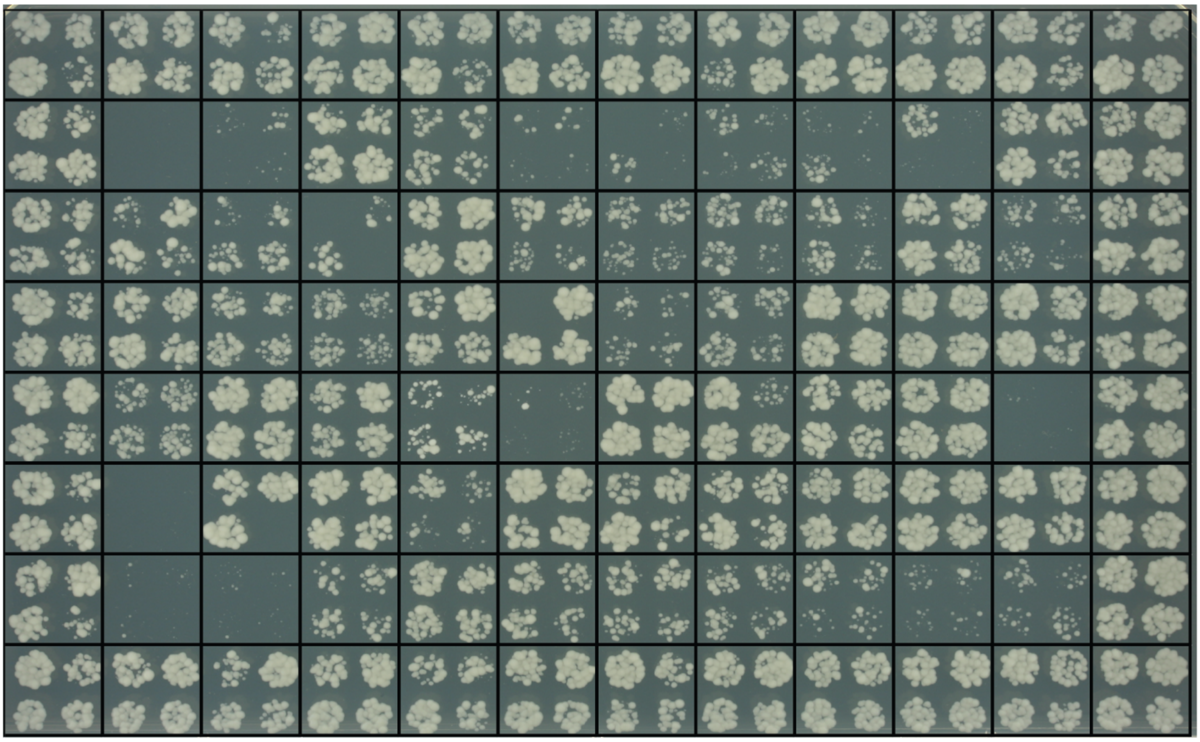

Figure S1 60 *yfgΔ est1Δ* strains arrayed in quadruplicate on a 384 format plate. (A) Strain map indicating the position of 60 genotypes on the 384 format plate in panel B; four replicates drawn in light grey. Red, purple, and green names indicate the reason for choosing particular genes for this study. (B) Photograph of a 384 format plate at passage 1 (48 hours after inoculation) from the liquid procedure (Figure 1A)
