# Supplementary material for: Genome-Wide Analysis to Identify Pathways Affecting Telomere-Initiated Senescence in Budding Yeast
Source: G3 (Bethesda). 2011 Aug 1;1(3):197–208. doi: 10.1534/g3.111.000216 (PMC3276134; doi:10.1534/g3.111.000216)
Supplement: Supporting Information [file supp_1.3.197_FigureS2.pdf]

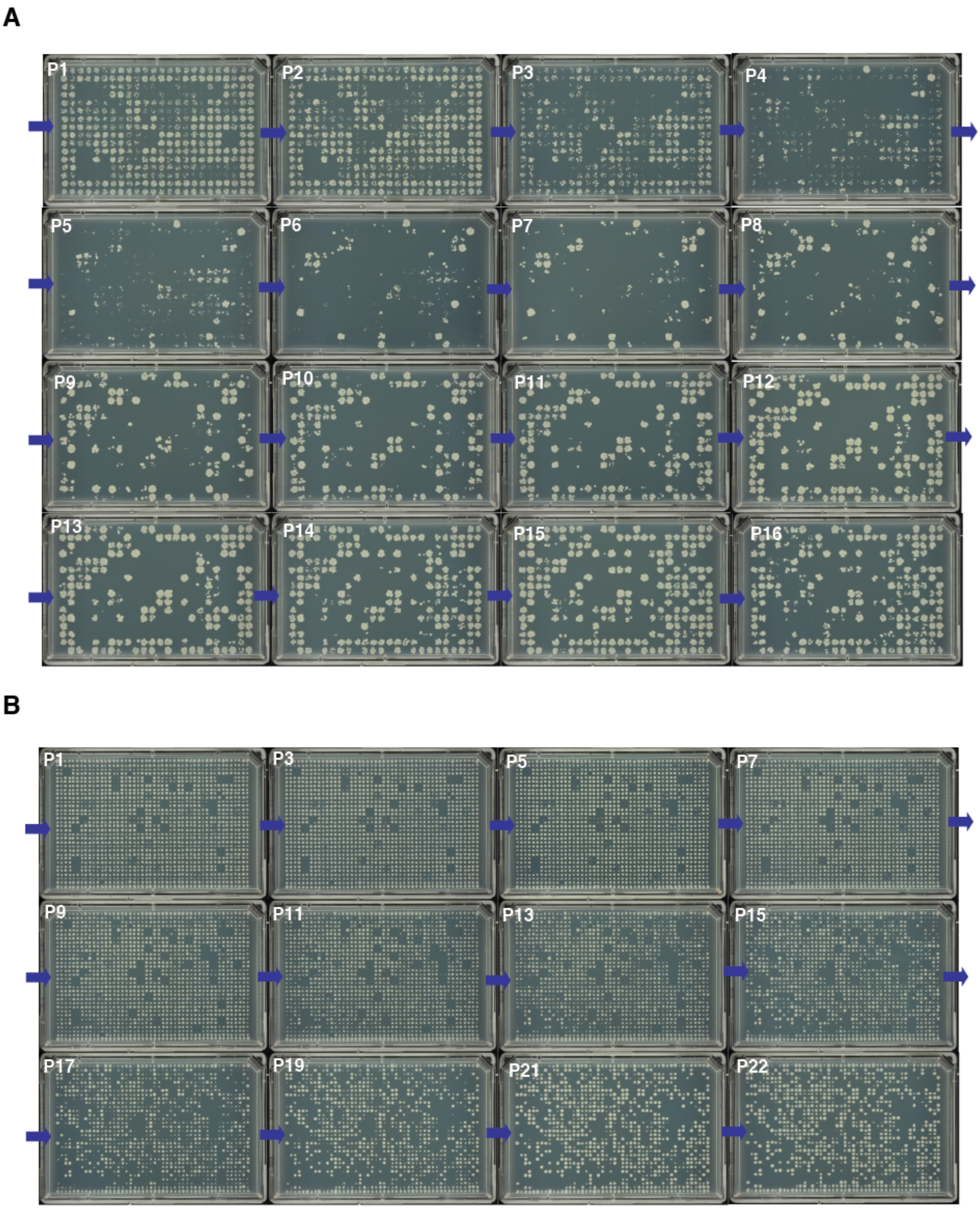

Figure S2 Example photographs from passaged cultures. (A) Photographs of 384 format agar plates for each of the 16 passages from the liquid procedure. (B) 12 sample photographs at various passages (indicated) for of one of the 1536 format plates from the solid procedure. Passage numbers are labelled on each photograph; P1-passage 1, etc.
