# Supplementary material for: Genome-Wide Analysis to Identify Pathways Affecting Telomere-Initiated Senescence in Budding Yeast
Source: G3 (Bethesda). 2011 Aug 1;1(3):197–208. doi: 10.1534/g3.111.000216 (PMC3276134; doi:10.1534/g3.111.000216)
Supplement: Supporting Information [file supp_1.3.197_FigureS3.pdf]

A

	P1	P2	P3	P4	P5	P6	P7	P8	P9	P10	P11	P12	P13
**WT**	314	330	334	344	330	384	460	442	436	372	286	610	400
est3Δ	369	240	137	15	7	29	510	282	362	482	292	378	399
est1Δ	410	269	143	23	9	44	521	301	332	387	297	400	365
est3Δ est1Δ	212	120	85	19	76	110	310	230	305	386	255	388	303

B

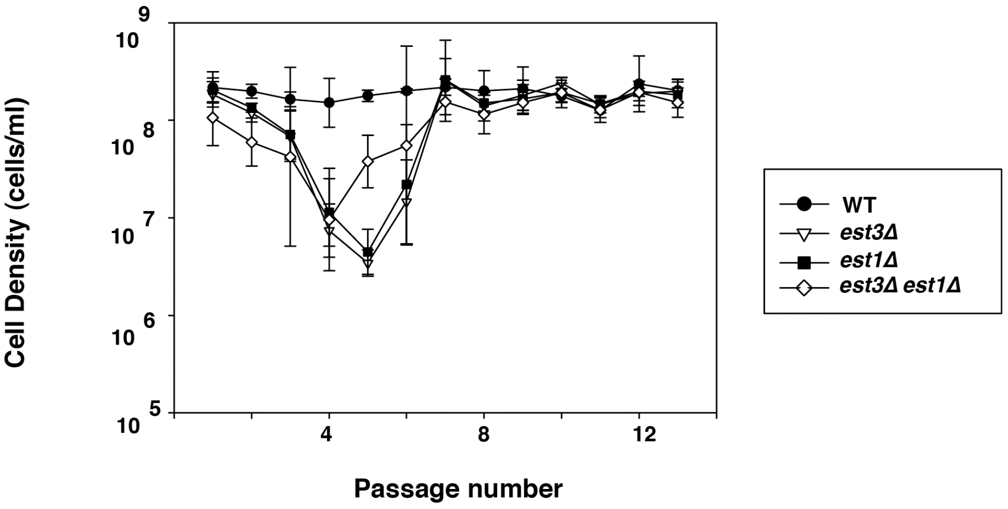

Figure S3 Low through-put senescence experiment in the W303 genetic background. (A) Six independent strains of each genotype in the W303 background were taken directly from germination plates and grown in liquid culture. Cell densities were counted every 23 hrs, followed by dilution to 5×10^5 cells/ml and continued incubation. Cell density reached by cells with the same genotype after 23 hours were recorded and averaged. The dilution factors at each passage were calculated and showed in the table, genotypes are indicated. (B) The data from (A) were plotted. Each symbol represents the average cell density reached by cells with the same genotype after 23 hours and the error bars represent the standard deviations.
