# Supplementary material for: Genome-Wide Analysis to Identify Pathways Affecting Telomere-Initiated Senescence in Budding Yeast
Source: G3 (Bethesda). 2011 Aug 1;1(3):197–208. doi: 10.1534/g3.111.000216 (PMC3276134; doi:10.1534/g3.111.000216)
Supplement: Supporting Information [file supp_1.3.197_FigureS4.pdf]

A

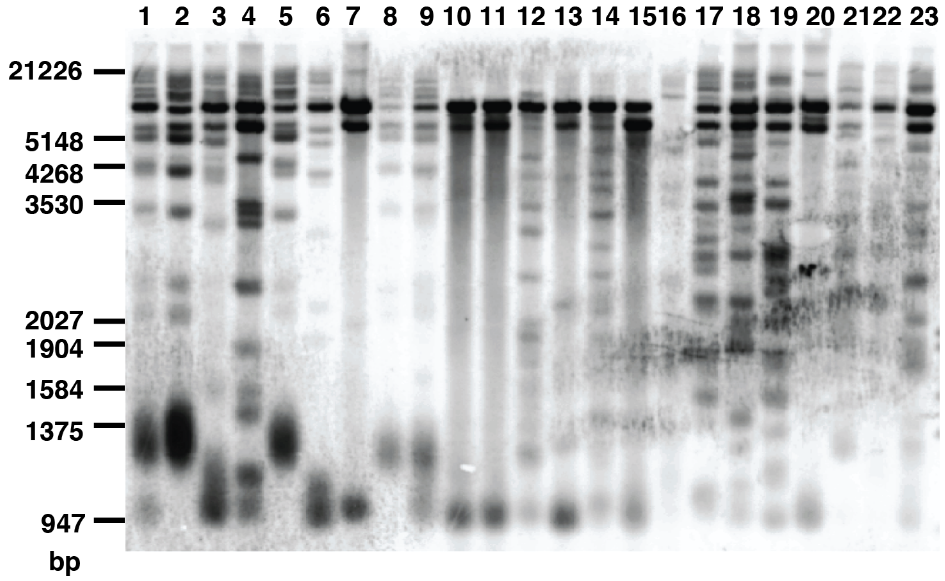

1. Wild type (DLY3799)
2. *sgs1Δ*
3. *sgs1Δ est1Δ* P1
4. *sgs1Δ est1Δ* P22
5. *rad17Δ*
6. *rad17Δ est1Δ* P1
7. *rad17Δ est1Δ* P22
8. *his3Δ*
9. *his3Δ est1Δ* P1
10. *his3Δ est1Δ* P22
11. *dcc1Δ est1Δ* P22
12. *cdc73Δ est1Δ* P22
13. *exo1Δ est1Δ* P22
14. *ebs1Δ est1Δ* P22
15. *upf3Δ est1Δ* P22
16. *rad27Δ est1Δ* P22
17. *ctf18Δ est1Δ* P22
18. *rif1Δ est1Δ* P22
19. *rad9Δ est1Δ* P22
20. *rif2Δ est1Δ* P22
21. *sae2Δ est1Δ* P22
22. *tel1Δ est1Δ* P22
23. *chk1Δ est1Δ* P22

P1-passage1
P22-passage22

Figure S4 Survivors were produced by passage 22 in the solid procedure. Genomic DNA prepared from the strains of different genotypes as indicated, digested with *XhoI* and subjected to Southern blot to detect telomeric Y' and "TG" fragments.
