# Supplementary material for: Genome-Wide Analysis to Identify Pathways Affecting Telomere-Initiated Senescence in Budding Yeast
Source: G3 (Bethesda). 2011 Aug 1;1(3):197–208. doi: 10.1534/g3.111.000216 (PMC3276134; doi:10.1534/g3.111.000216)
Supplement: Supporting Information [file supp_1.3.197_FigureS5.pdf]

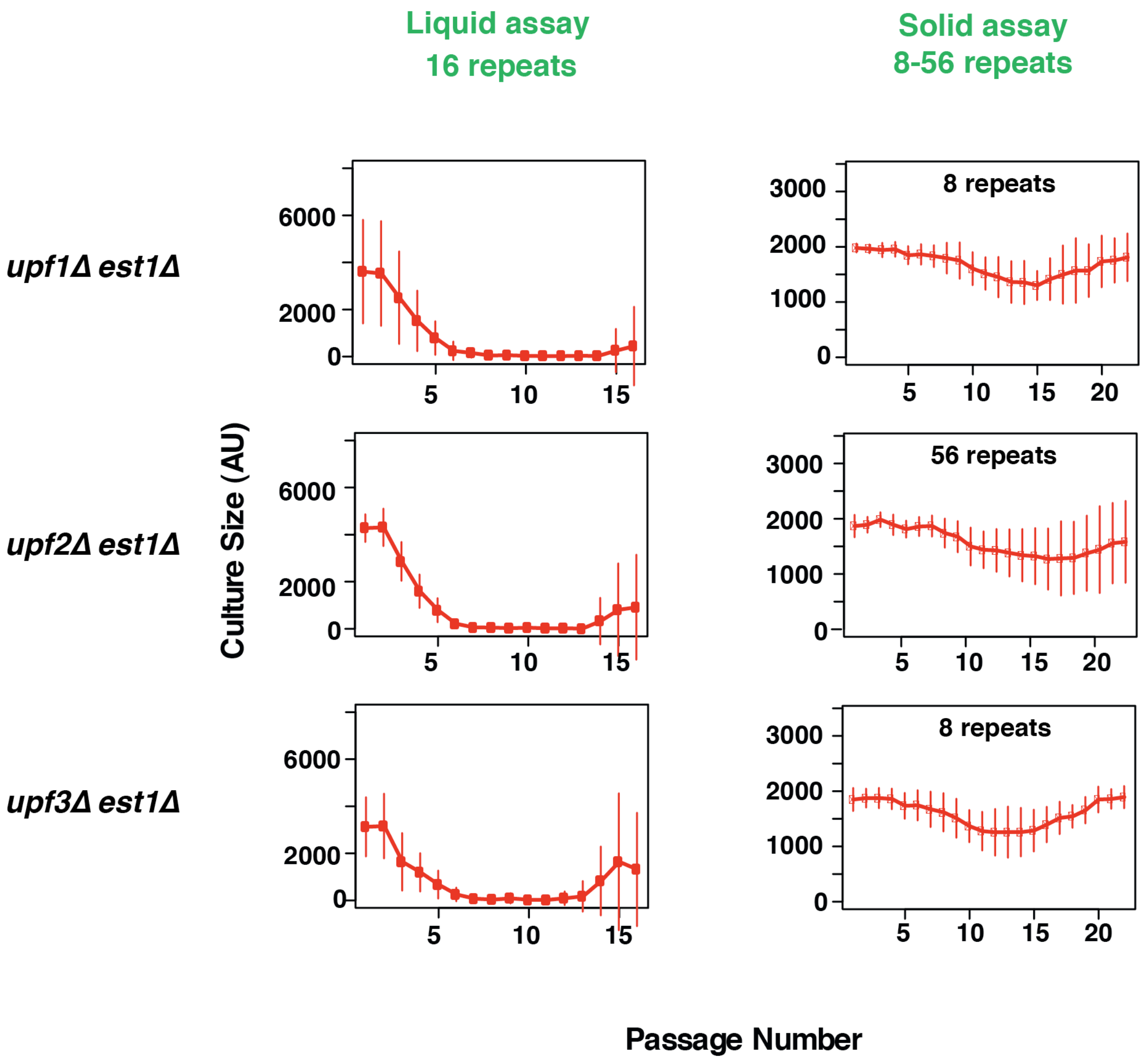

Figure S5 Nonsense mediated decay genes had consistent MDPs in both screens. *UPF1, UPF2,* and *UPF3* gene deletions had consistent MDPs patterns in both the liquid procedure and in the solid procedure. Replicate number and experiment type are indicated in green text.
