# Supplementary material for: Genome-Wide Analysis to Identify Pathways Affecting Telomere-Initiated Senescence in Budding Yeast
Source: G3 (Bethesda). 2011 Aug 1;1(3):197–208. doi: 10.1534/g3.111.000216 (PMC3276134; doi:10.1534/g3.111.000216)
Supplement: Supporting Information [file supp_1.3.197_FigureS6.pdf]

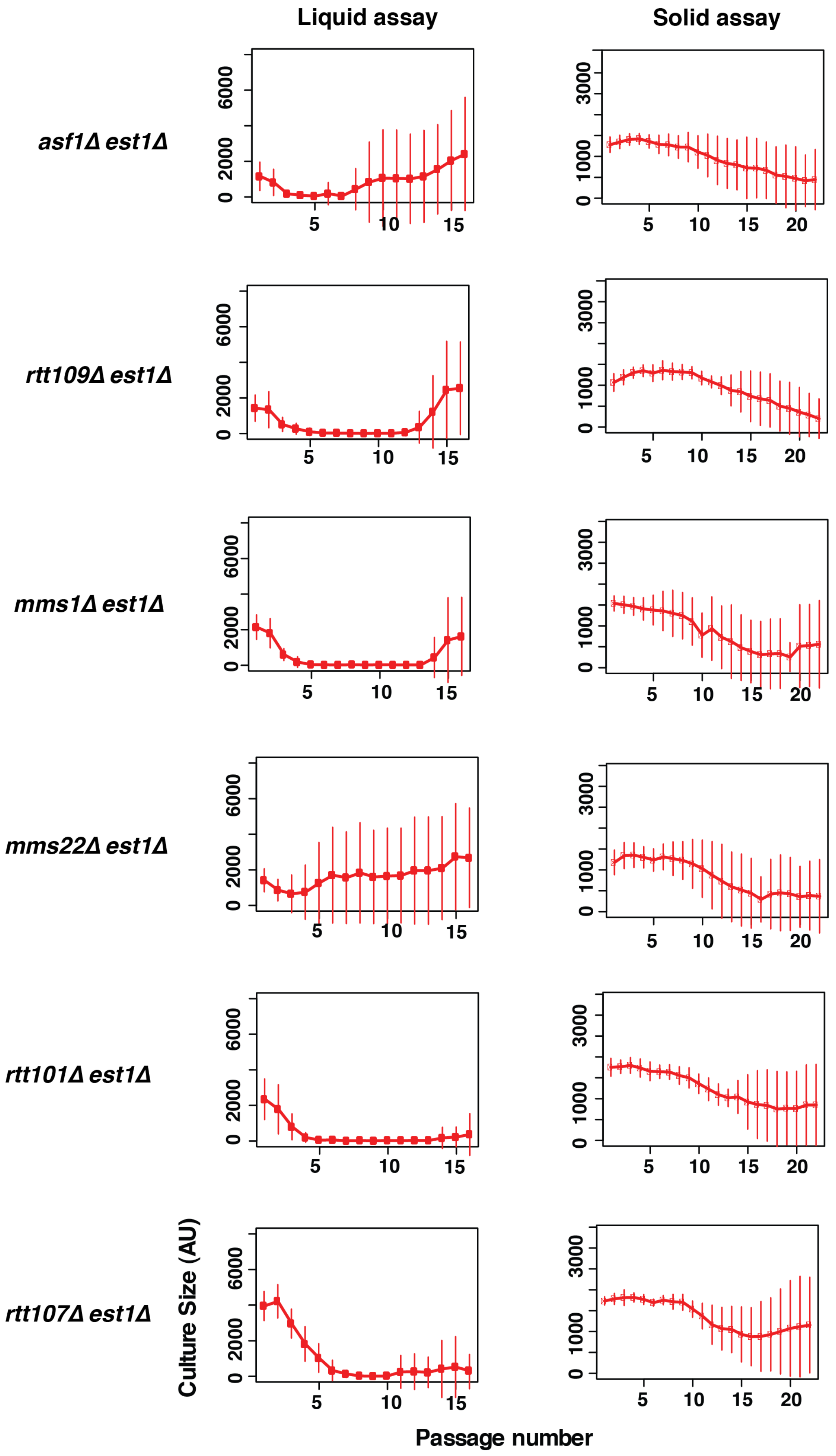

Figure S6 Genes affecting replication fork progression had an accelerated senescence phenotype when deleted in the *est1Δ* background. MDPs for *asf1Δ est1Δ*, *rtt109Δ est1Δ*, *mms1Δ est1Δ*, *mms22Δ est1Δ*, *rtt101Δ est1Δ*, and *rtt107Δ est1Δ* indicating a fast senescence phenotype in both the liquid and solid assay.
