# Supplementary material for: Genome-Wide Analysis to Identify Pathways Affecting Telomere-Initiated Senescence in Budding Yeast
Source: G3 (Bethesda). 2011 Aug 1;1(3):197–208. doi: 10.1534/g3.111.000216 (PMC3276134; doi:10.1534/g3.111.000216)
Supplement: Supporting Information [file supp_1.3.197_FigureS7.pdf]

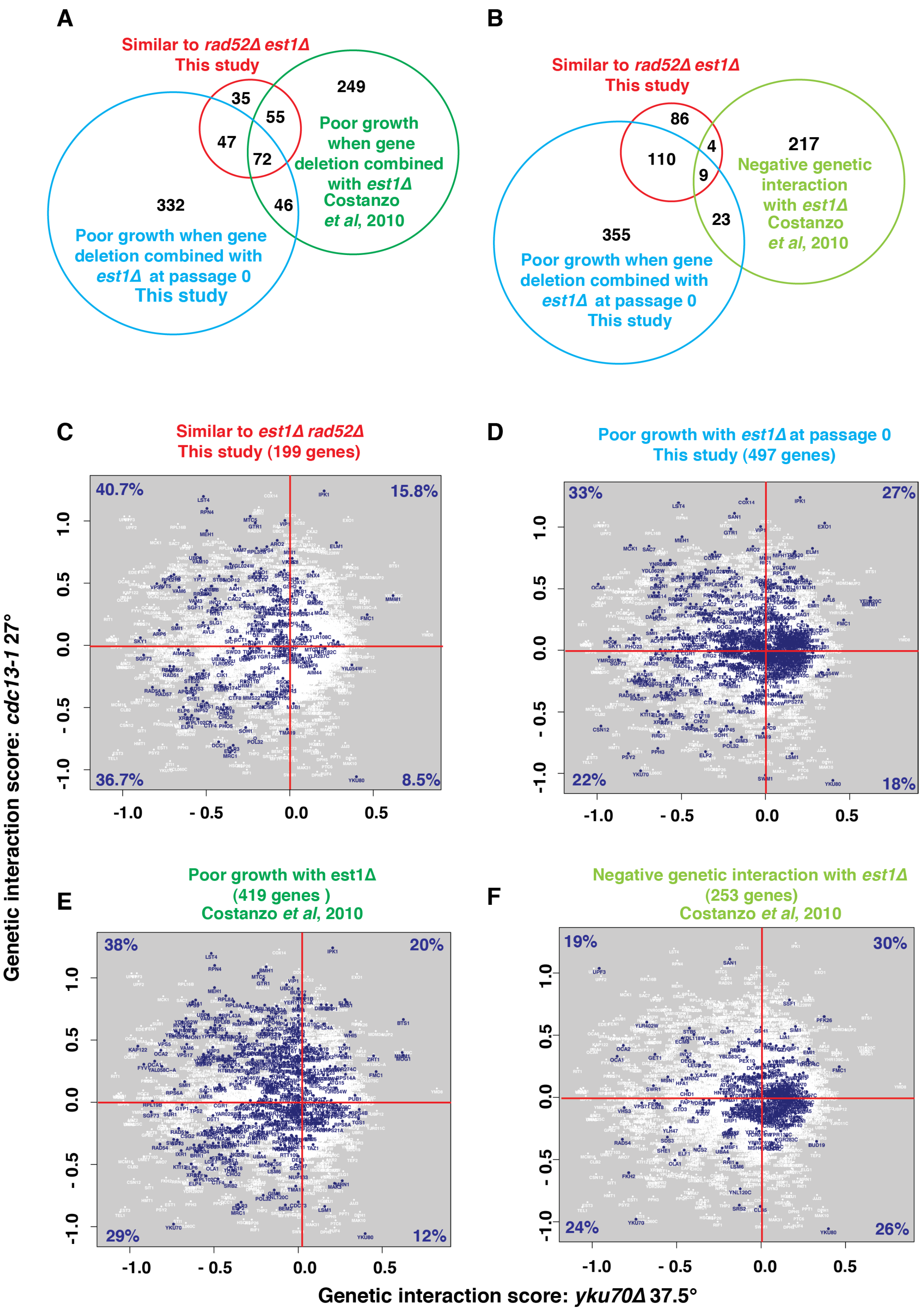

Figure S7 Repeated passage of cultures provides useful information about telomere-dependent senescence. (A) Venn diagram demonstrating the overlap between poor growers from our initial SGA (blue circle), poor growers from the *est1Δ* SGA by (Costanzo *et al.* 2010b) (green circle) and our accelerated senescence class defined by repeated passaging, similar to *rad52Δ*, Figure 4B, Supporting File S7 (red circle). Poor growers are defined here as deletions whose fitness is more than one standard deviation less than the mean fitness across all deletions in the library. (B) As for panel A,

except the green circle in this panel represents genes classified as having a lenient negative interaction with *est1Δ* by Costanzo et al. (C) *yku70Δ* vs *cdc13-1* genetic interaction profile (see Figure 6) with our accelerated senescence class overlaid (blue). (D) *yku70Δ* vs *cdc13-1* genetic interaction profile with poor growers from our initial SGA overlaid in blue (E) *yku70Δ* vs *cdc13-1* genetic interaction profile with poor growers from the *est1Δ* SGA by Costanzo et al. overlaid (blue) (F) *yku70Δ* vs *cdc13-1* genetic interaction profile with genes classified as having a lenient negative interaction with *est1Δ* by Costanzo et al. overlaid (blue, see Figure 4A, Table ST4).
