# Supplementary material for: Genome-Wide Analysis to Identify Pathways Affecting Telomere-Initiated Senescence in Budding Yeast
Source: G3 (Bethesda). 2011 Aug 1;1(3):197–208. doi: 10.1534/g3.111.000216 (PMC3276134; doi:10.1534/g3.111.000216)
Supplement: Supporting Information [file supp_1.3.197_FigureS8.pdf]

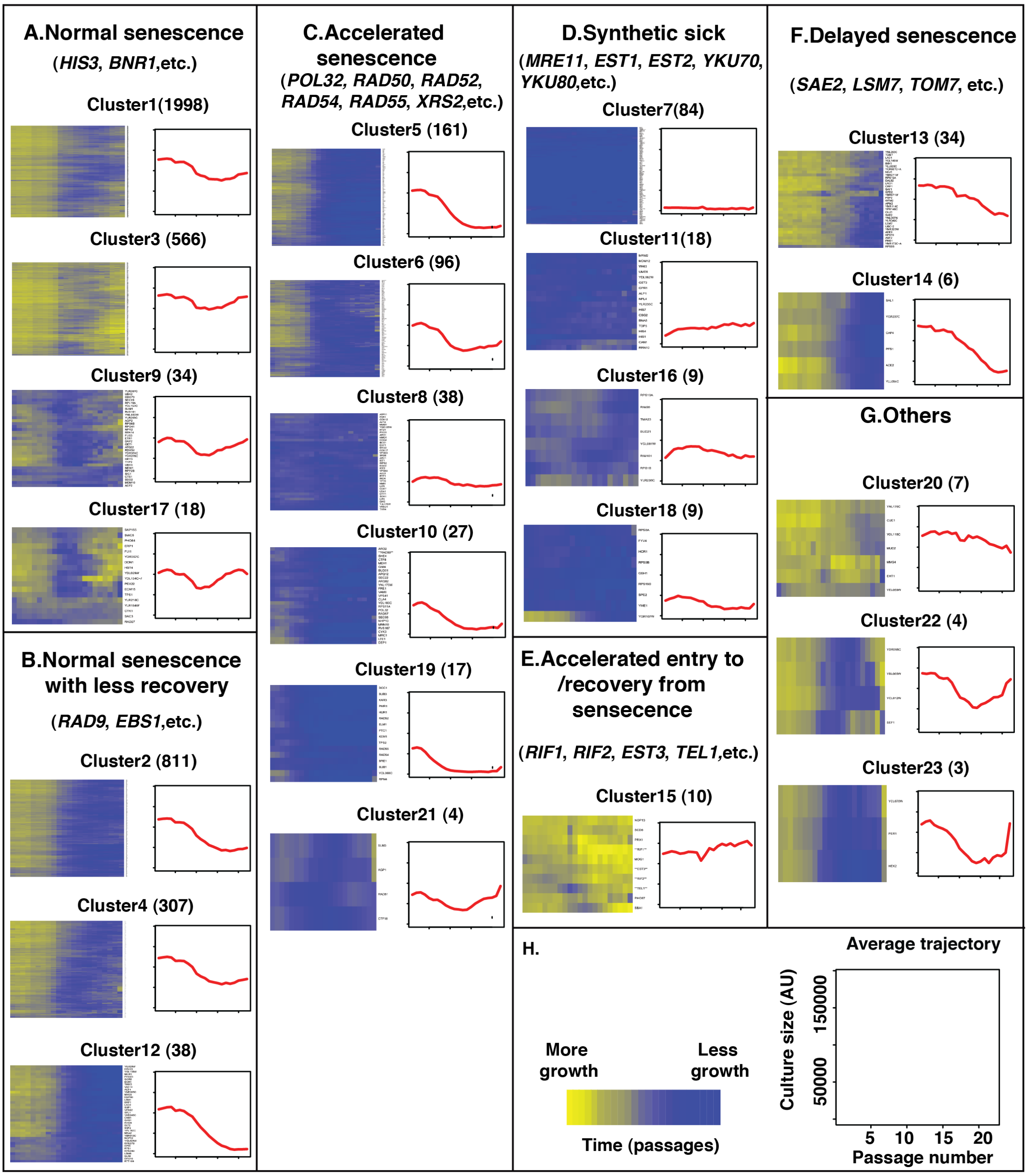

Figure S8 Unsupervised hierarchical QT clustering identifies 23 different MDP classes. Heat map representation of cluster MDPs on left, average MDPs on right. 23 automatically generated classes were combined manually into 8 functionally meaningful classifications (A-H).
