# Supplementary material for: Genome-Wide Analysis to Identify Pathways Affecting Telomere-Initiated Senescence in Budding Yeast
Source: G3 (Bethesda). 2011 Aug 1;1(3):197–208. doi: 10.1534/g3.111.000216 (PMC3276134; doi:10.1534/g3.111.000216)
Supplement: Supporting Information [file supp_1.3.197_Files.pdf]

Files S1-S13

All supporting files listed below (together with raw images and initial culture size quantifications) can be downloaded from our Supplemental Data website: http://research.ncl.ac.uk/colonyzer/ChangSenescence/

File S1. Liquid screen MDP plots.
Mean Density Profiles for each of 60 strains grown in the liquid culture screen. Red points are average culture size for all available repeats at passages 1 - 16. Vertical red lines show standard deviation for all repeats.

File S2. Solid screen MDP plots.
Mean Density Profiles for each of ~4300 strains grown in the solid agar screen. Red points are average culture size for all available repeats at passages 1 - 22. Vertical red lines show standard deviation for all repeats.

File S3. Manual classification of MDPs from liquid screen.
Manual functional clustering of the 60 gene deletions investigated in the liquid culture screen. MDPs for each cluster member plotted in panels A – P. 3 main categories are indicated in black text; functionally related sub-categories are defined by panels and titled in green text: (A) Recombination repair (B) *CTF18* RFC-like complex (C) Nonsense mediated decay. (D) Histone acetyltransferase. (E) MRX complex. (F) *SLX5-SLX8* STUbL Complex. (G) Ubiquitin-conjuating enzyme. (H) AF1 complex. (I) *SUM1/RFM1* repressor complex. (J) Mean plots of gene deletions that accelerate senescence. (K) Mean plots of gene deletions that accelerated entry to and escaped from senescence. (L) Subunit of N-terminal acetyltrasferase. (M) DNA damage checkpoint. (N) 14-3-3 proteins. (O&P) Gene deletions that do not affect senescence. Dashed lines indicate genes that are not classified in any particular group.

File S4. MDPs for all 4299 strains in the solid screen.
Tab-delimited text file containing quantitative MDPs (culture size profiles with increasing passage). Culture sizes are in arbitrary units. Note that photo 1 corresponds to the first photograph taken after the SGA. Passage 1 in the manuscript corresponds to photo 2.

File S5. MDPs similar to that of the *his3Δ est1Δ* archetype from the solid screen.
Tab-delimited text file listing Pearson's correlation coefficient (c) between the MDP of the archetype strain *his3Δ est1Δ* and all other deletions in the library. Gene deletions are ranked by correlation coefficient and thereby profile similarity. Root Mean Square difference (RMS) between the MDP of each strain and that of the archetype is also reported, together with an estimate of the area under the MDP of each strain (Integral).

File S6. MDPs similar to that of the *rad52Δ est1Δ* archetype from the solid screen.
As for File S5.

File S7. MDPs similar to that of the *rad52Δ est1Δ* archetype from the solid screen which are also poor growers.
As for S5, except similarity is defined as deletions in the library with Corr > 0.5 and Culture Size at passage 2 < 1680 (see Figure 4).

File S8. MDPs similar to that of the *rif1Δ est1Δ* archetype from the solid screen.
As for File S5.

File S9. Gene lists used in the Venn diagram in Figure S7
Tab-delimited lists of gene names classified as having similar MDPs to the *rad52Δ est1Δ* archetype (RAD52Archetype), classified as being poor growers after the SGA presented in this work (SGASick), classified as being poor growers in the *est1Δ* double mutant fitness data set Costanzo et al. 2010 (Supplementary data file S1) (CostanzoSick) or classified as having a (lenient) negative interaction with *est1Δ* by Costanzo et al. 2010 (CostanzoNegGIS). Poor growers were defined as having a fitness or culture size less than the mean by more than one standard deviation.

File S10. Intersection classes in Venn diagram Figure S7.
Venn diagram from Figure S7 repeated with letters classifying groups of gene deletions from overlapping gene lists as defined in File S9.

File S11. Gene members of interaction classes in Venn diagram in File S10.
Tab-delimited member lists for gene deletion categories defined in File S10.

File S12. 23 clusters from unsupervised QT clustering of MDPs from solid screen.
Clusters are represented by MDP heat maps showing culture size variation with passage (V1-V22), dendograms linking MDPs (left) the names of gene deletions for each MDP (right) together with alternating plots of the average MDP for all members of each cluster (red curve, passage number on x-axis, culture size on y-axis).

File S13. Gene members of clusters presented in File S12
Tab-delimited text file classifying each ORF in the deletion library by cluster membership.
