# Supplementary material for: Genome-Wide Analysis to Identify Pathways Affecting Telomere-Initiated Senescence in Budding Yeast
Source: G3 (Bethesda). 2011 Aug 1;1(3):197–208. doi: 10.1534/g3.111.000216 (PMC3276134; doi:10.1534/g3.111.000216)
Supplement: Supporting Information [file supp_1.3.197_TableS1.pdf]

Table S1 60 genes tested by liquid senescence assay

Gene	*Senescence phenotype	**Telomere length screens	Gene ontology annotation	***References
EST3	AERS	VS	telomerase component	*est2Δ est3Δ/est1Δ est3Δ/tlc1Δ est3Δ* showed normal senescence (Lendvay *et al.* 1996)
RIF1	AERS	VL	telomere binding protein	*rif1Δ est2Δ* showed normal senescence (Anbalagan S *et al.* 2011); *rif1Δ est2Δ* accelerated entry to /recovery from senescence (Chang *et al.* 2011)
RIF2	AERS	VL	telomere binding protein	*rif2Δ est2Δ* accelerated entry to/recovery from senescence (Chang *et al.* 2011)
EST1	AS	VS	telomerase component	
MRE11	AS	VS	DNA repair/ MRX complex	Normal (Le *et al.* 1999)
XRS2	AS	VS	DNA repair/MRX complex	Normal (Le *et al.* 1999)
RAD52	AS		recombinational repair of double-strand breaks in DNA	*rad52Δ est1Δ and rad52Δ tlc1Δ* accelerated senescence (Abdallah *et al.* 2010; Le *et al.* 1999; LeBel *et al.* 2009; Lee *et al.* 2007; Lundblad and Blackburn 1993)
RAD54	AS		recombinational repair of double-strand breaks in DNA	*rad54Δ tlc1Δ* has severe growth defect (Le *et al.* 1999)
RAD55	AS		recombinational repair of double-strand breaks in DNA	
RAD57	AS		recombinational repair of double-strand breaks in DNA	*rad57Δ tlc1Δ* has severe growth defect (Le *et al.* 1999)
UPF1 (NAM7)	AS	VS	nonsense mediated deccay	*upf1Δ* with *tlc1Δ/est1Δ/est2Δ/est3Δ* delayed senescence measured by a different method (Enomoto *et al.* 2004)
UPF2 (NMD2)	AS	S	nonsense mediated deccay	*upf2Δ* with *tlc1Δ/est1Δ/est2Δ/est3Δ* delayed senescence measured by a different method (Enomoto *et al.* 2004)
UPF3	AS	S	nonsense mediated deccay	*upf3Δ* with *tlc1Δ/est1Δ/est2Δ/est3Δ* delayed senescence measured by a different method (Enomoto *et al.* 2004)

DCC1	AS	ss	Sister chromatid cohesion	
CTF18	AS	Literature reported	Sister chromatid cohesion	
CTF8	AS	ss	Sister chromatid cohesion	
ELG1	AS	L	RFC complex	
RTT101	AS		Histone acetyltransferase/involved in NHEJ	
RTT109	AS		Histone acetyltransferase/involved in NHEJ	
RTT107	AS		Mms22-dependent DNA repair during S phase/interacts with Mms22p and Slx4p	
MMS1	AS		Subunit of an E3 ubiquitin ligase complex/resolving replication intermediates	*mms1Δ tlc1Δ* accelerated senescence and failed to recover (ABDALLAH *et al.* 2010)
MMS22	AS		Ubiquitin-conjugating enzyme variant involved in error-free post replication repair	
CDC73	AS	S	PAF1 complex	
RTF1	AS	ss	PAF1 complex	
SLX8	AS	sl,	Subunit of the Slx5-Slx8 SUMO-targeted ubiquitin ligase (STUbL) complex	*slx8Δ tlc1Δ* accelerated senescence (AZAM *et al.* 2006)
SLX5 (HEX3)	AS		Subunit of the Slx5-Slx8 SUMO-targeted ubiquitin ligase (STUbL) complex	*slx5Δ tlc1Δ* accelerated senescence (AZAM *et al.* 2006)

RRP8	AS	L	pre-rRNA processing/methyltransferase	
SPT21	AS	ss	regulator of histone gene transcription	
POL32	AS	sl	Polymerase delta subunit	*pol32Δ tlc1Δ* accelerated senescence (Lydeard *et al.* 2007)
SGS1	AS		Nucleolar DNA helicase of the RecQ family	*sgs1Δ tlc1Δ* accelerated senescence (Azam *et al.* 2006; Lee *et al.* 2007)
RAD27	AS	VL	flap-endonuclease	*rad27Δ* with *est1Δ/tlc1Δ/est3Δ/cdc13-2* accelerated senescence (Parenteau and Wellinger 2002)
HMO1	AS	L	HMG-box protein	
TEL1	AS	VS	PIK homologue	*tel1Δ tlc1Δ showed* normal senescence (Enomoto *et al.* 2002) ; *tel1Δ tlc1Δ* delayed senescence (Ritchie *et al.* 1999), *tel1Δ tlc1Δ mec1Δ sml1Δ* is normal (Chan *et al.* 2001); *tel1Δ tlc1Δ* delayed senescence (Abdallah *et al.* 2010)
KEM1	AS	S	RNA degradation	
YDL118	AS	ss	Unknown	
LEA1	AS	L	RNA splicing	
SUM1	AS	S	SUM1/RFM1 repressor complex	
RFM1	AS	S	SUM1/RFM1 repressor complex	
TSA1	AS		Thioredoxin peroxidase/ribosome-associated and free cytoplasmic antioxidant	
ASF1	AS		Nucleosome assembly factor/chromatin assembly and disassembly	
SLA1	AS		Cytoskeletal binding protein	

RHO4	AS		Non-essential small GTPase of the Rho/Rac subfamily of Ras-like proteins	
EDE1	AS		Key endocytic protein	
EXO1	Normal	Literature reported	5'-3' exonuclease and flap-endonuclease	*exo1Δ tlc1Δ* delayed senescence (Maringele and Lydall 2004)
EBS1	Normal	ss	nonsense mediated decay	
RAD9	Normal	Literature reported	DNA damage checkpoint effector	*rad9Δ tlc1Δ* had less G2/M arrested cells (Ijpma and Greider 2003)
RAD17	Normal	Literature reported	DNA damage checkpoint effector	
DDC1	Normal	Literature reported	DNA damage checkpoint effector	
RAD24	Normal	Literature reported	DNA damage checkpoint effector	*rad24Δ tlc1Δ* had less G2/M arrested cells (Ijpma and Greider 2003)
CHK1	Normal		DNA damage checkpoint effector	
BMH1	Normal	Literature reported	14-3-3 protein	
BMH2	Normal	Literature reported	14-3-3 protein	
MRPL44	Normal	ss	mitochondrial ribosomal protein	
MOT3	Normal	ss	POLII transcription	
HST3	Normal		Member of the Sir2 family of NAD(+)-dependent protein deacetylases	
CSM3	Normal		Replication fork associated factor	
BNR1	Normal		Formin	
HIS3	Normal		Imidazoleglycerol-phosphate dehydratase	Control strain
MAK3	Normal	L	N-terminal acetyltransferase complex	
MAK10	Normal	L	N-terminal acetyltransferase complex	
MAK31	Normal	L	N-terminal acetyltransferase complex	

Legend: Examining of the 60 genes selected for the liquid procedure in this study. The references quoted were indicated in the table and included below.

*Senescence phenotype abbreviations: AS- accelerated senescence, AERS-accelerated entry to and recovery from senescence.
Normal: normal entry to and recovery from senescence.
** Telomere phenotype abbreviations (Askree *et al.* 2004; Gatbonton *et al.* 2006; Shachar *et al.* 2008): VS - very short, S - short, ss - slightly short, sl - slightly long, L - long, VL - very long.
*** Conflicts in data may be due to differences in ways senescence was measured.

References

Abdallah, P., P. Luciano, K. W. Runge, M. Lisby, V. Geli *et al.*, 2010 A two-step model for senescence triggered by a single critically short telomere (vol 11, pg 988, 2009). Nature Cell Biology **12:** 520-520.

Anbalagan S, Bonetti D, Lucchini G and L. MP., 2011 Rif1 Supports the Function of the CST Complex in Yeast Telomere Capping. PLos Genetics **7**.

Askree, S. H., T. Yehuda, S. Smolikov, R. Gurevich, J. Hawk *et al.*, 2004 A genome-wide screen for Saccharomyces cerevisiae deletion mutants that affect telomere length. Proceedings of the National Academy of Sciences of the United States of America **101:** 8658-8663.

Azam, M., J. Y. Lee, V. Abraham, R. Chanoux, K. A. Schoenly *et al.*, 2006 Evidence that the S.cerevisiae Sgs1 protein facilitates recombinational repair of telomeres during senescence. Nucleic Acids Research **34:** 506-516.

Chan, S. W. L., J. Chang, J. Prescott and E. H. Blackburn, 2001 Altering telomere structure allows telomerase to act in yeast lacking ATM kinases. Current Biology **11:** 1240-1250.

Chang, M., J. C. Dittmar and R. Rothstein, 2011 Long telomeres are preferentially extended during recombination-mediated telomere maintenance. Nature structural and molecular biology.

Enomoto, S., L. Glowczewski and J. Berman, 2002 MEC3, MEC1, and DDC2 are essential components of a telomere checkpoint pathway required for cell cycle arrest during senescence in Saccharomyces cerevisiae. Molecular Biology of the Cell **13:** 2626-2638.

Enomoto, S., L. Glowczewski, J. Lew-Smith and J. G. Berman, 2004 Telomere cap components influence the rate of senescence in telomerase-deficient yeast cells. Molecular and Cellular Biology **24:** 837-845.

Gatbonton, T., M. Imbesi, M. Nelson, J. M. Akey, D. M. Ruderfer *et al.*, 2006 Telomere length as a quantitative trait: Genome-wide survey and genetic mapping of telomere length-control genes in yeast. Plos Genetics **2:** 304-315.

Ijpma, A. S., and C. W. Greider, 2003 Short telomeres induce a DNA damage response in Saccharomyces cerevisiae. Molecular Biology of the Cell **14:** 987-1001.

Le, S., J. K. Moore, J. E. Haber and C. W. Greider, 1999 RAD50 and RAD51 define two pathways that collaborate to maintain telomeres in the absence of telomerase. Genetics **152:** 143-152.

LeBel, C., E. Rosonina, D. C. F. Sealey, F. Pryde, D. Lydall *et al.*, 2009 Telomere Maintenance and Survival in Saccharomyces cerevisiae in the Absence of Telomerase and RAD52. Genetics **182:** 671-684.

Lee, J. Y., M. Kozak, J. D. Martin, E. Pennock and F. B. Johnson, 2007 Evidence that a RecQ helicase slows senescence by resolving recombining telomeres. Plos Biology **5:** 1334-1344.

Lendvay, T. S., D. K. Morris, J. Sah, B. Balasubramanian and V. Lundblad, 1996 Senescence mutants of Saccharomyces cerevisiae with a defect in telomere replication identify three additional EST genes. Genetics **144:** 1399-1412.

Lundblad, V., and E. H. Blackburn, 1993 AN ALTERNATIVE PATHWAY FOR YEAST TELOMERE MAINTENANCE RESCUES EST1-SENESCENCE. Cell **73:** 347-360.

Lydeard, J. R., S. Jain, M. Yamaguchi and J. E. Haber, 2007 Break-induced replication and telomerase-independent telomere maintenance require Pol32. Nature **448:** 820-U810.

Maringele, L., and D. Lydall, 2004 EXO1 plays a role in generating type I and type II survivors in budding yeast. Genetics **166:** 1641-1649.

Parenteau, J., and R. J. Wellinger, 2002 Differential processing of leading- and lagging-strand ends at Saccharomyces cerevisiae telomeres revealed by the absence of Rad27p nuclease. Genetics **162:** 1583-1594.

Ritchie, K. B., J. C. Mallory and T. D. Petes, 1999 Interactions of TLC1 (which encodes the RNA subunit of telomerase), TEL1, and MEC1 in regulating telomere length in the yeast Saccharomyces cerevisiae. Molecular and Cellular Biology **19:** 6065-6075.

Shachar, R., L. Ungar, M. Kupiec, E. Ruppin and R. Sharan, 2008 A systems-level approach to mapping the telomere length maintenance gene circuitry. Molecular Systems Biology **4**.
