# Supplementary material for: Genome-Wide Analysis to Identify Pathways Affecting Telomere-Initiated Senescence in Budding Yeast
Source: G3 (Bethesda). 2011 Aug 1;1(3):197–208. doi: 10.1534/g3.111.000216 (PMC3276134; doi:10.1534/g3.111.000216)
Supplement: Supporting Information [file supp_1.3.197_TableS2.pdf]

Table S2 Gene ontology analysis (processes) of RAD52-like genes from the solid screen in this study

GO-Slim term	Genes annotated to the term
Transport	*AGP1, APQ12, ATG9, AVL9, AVT5, CCZ1, CIK1, CLA4, DRS2, FKS1, GCS1, GET2, GOS1, GTR1, KAR3, LST4, MMM1, NUM1, PEX12, PEX14, PEX19, PEX5, PMR1, RIM8, RPS18B, SEC28, SEC66, SGF73, SKY1, SLG1, SNX4, SPC2, SPF1, SSO2, STE20, THR4, TIM18, TLG2, UBP3, UBX2, VAM3, VAM6, VAM7, VPS1, VPS4, VPS41, VPS5, VPS51, VTH1, YDL183C, YPT7, ZRC1*
Response to stress	*ASF1, ATG9, BRE1, CAC2, CCZ1, CKB1, CTF18, CTF4, CTF8, DCC1, ELM1, GSH1, HDA1, MGA2, MMS1, MMS22, MRC1, MRE11, NHP10, NPL4, OPI1, PHO5, PHO80, POL32, RAD50, RAD52, RAD54, RAD55, RAD57, RMI1, RPN4, RTT109, SIC1, SIN3, SLG1, SLX5, SLX8, SNF6, SOH1, STB5, STE20, TAX4, TIM18, TMA19, TPS2, UBI4, URM1, VAM3, VPS75, XRS2, YKU80*
RNA metabolic process	*ASF1, BRE1, CAC2, CKB1, DEG1, ELP2, ELP3, ELP4, ELP6, GRS1, GTR1, HAP4, HDA1, HTZ1, INO2, IPK1, MED1, MGA2, MMS1, MRC1, MRE11, NCS6, NOP12, OPI1, PHO80, POL32, RPL35B, RPN4, RPS11B, RPS16A, RPS18B, RTF1, RTT109, SDS3, SGF11, SGF73, SIN3, SKY1, SNF4, SNF6, SOH1, STB5, SWD1, SWD3, URM1, YGR122W, YKU80, YPL205C, ZDS2*
Chromosome organization	*ARP6, ASF1, BRE1, CAC2, CIK1, CTF18, CTF4, CTF8, DCC1, HDA1, KAR3, LGE1, MMS22, MRC1, NHP10, RAD50, RAD51, RAD52, RAD54, RAD57, RMI1, RTF1, RTS1, RTT109, SDS3, SGF11, SGF29, SGF73, SIN3, SLX5, SLX8, SNF6, SOH1, SWD1, SWD3, VPS75, XRS2, YKU80*
Transcription, DNA-dependent	*ASF1, BRE1, CAC2, CKB1, ELP2, ELP3, ELP4, ELP6, GRS1, GTR1, HAP4, HDA1, HTZ1, INO2, MED1, MGA2, MRC1, MRE11, OPI1, PHO80, RPN4, RTF1, RTT109, SDS3, SGF11, SGF73, SIN3, SNF4, SNF6, SOH1, STB5, SWD1, SWD3, YGR122W, YKU80, YPL205C, ZDS2*
Protein modification process	*ASF1, BRE1, CKB1, CLA4, DIA2, ELM1, ELP2, ELP6, HDA1, HPM1, LGE1, MUB1, NCS6, OST4, PHO80, RTF1, RTS1, RTT109, SDS3, SGF11, SGF29, SGF73, SIC1, SIN3, SKY1, SLX5, SLX8, SNF4, STE20, SWD1, SWD3, UBI4, UBP3, UBP6, URM1, VPS75*
DNA metabolic process	*BRE1, CAC2, CTF18, CTF4, CTF8, DCC1, DIA2, HUR1, LGE1, MMS1, MMS22, MRC1, MRE11, NHP10, POL32, RAD50, RAD51, RAD52, RAD54, RAD55, RAD57, RIM1, RPN4, RTT109, SIN3, SLX5, SLX8, SNF6, SOH1, SWD1, SWD3, VPS75, XRS2, YKU80*
Cell cycle	*BRE1, CIK1, CLA4, CTF18, CTF4, CTF8, DCC1, ELM1, FAR1, GCS1, KAR3, MMS22, MRC1, MRE11, PHO80, RAD50, RAD51, RAD52, RAD55, RAD57, RIM8, RMI1, RPN4, RTS1, SAP155, SHE1, SIC1, SIN3, SMI1, SOH1, STE20, XRS2, ZDS2*
Vesicle-mediated transport	*AVL9, CCZ1, DRS2, FKS1, GCS1, GET2, GOS1, LST4, PMR1, RIM8, SEC28, SLG1, SNX4, SSO2, THR4, TLG2, UBP3, VAM3, VAM6, VAM7, VPS1, VPS4, VPS41, VPS5, VPS51, VTH1, YPT7*
Cellular membrane organization	*APQ12, ATG9, CCZ1, DRS2, FKS1, GET2, GOS1, MEH1, MMM1, RIM8, SEC28, SLG1, SNX4, SSO2, THR4, TIM18, TLG2, VAM10, VAM3, VAM6, VAM7, VPS1, VPS4, VPS41, YPT7*
Biological process unknown	*AIM4, AIM44, IES2, IES5, MTC1, MTC5, PAR32, RTC2, YBR174C, YDR149C, YGL024W, YGL081W, YGR125W, YGR182C, YIL054W, YLR065C, YLR108C, YLR287C, YML012C-A, YNL170W, YPL102C*
Meiosis	*BRE1, CIK1, KAR3, MMS22, MRE11, RAD50, RAD51, RAD52, RAD55, RAD57, RIM8, RTS1, SOH1, XRS2*

Response to chemical stimulus	*CLA4, ELM1, FAR1, GSH1, NPL4, OPI1, RPN4, SKY1, STB5, STE20, TIM18, TMA19, URM1*
Protein complex biogenesis	*ATG9, ATP11, COX12, COX23, FMC1, GIM4, PEX14, PEX5, PKR1, SGF73, VAM6, VPS4, VPS41*
Cellular amino acid metabolic process	*ARO1, ARO2, GRS1, GSH1, GSH2, HOM6, PDC5, SPE1, SPE2, SPE3, THR4, ZRC1*
Cellular lipid metabolic process	*APQ12, CHO2, ETR1, INO2, INP52, MGA2, OAR1, OPI1, OPI3, PSD1, SCT1, TLG2*
Peroxisome organization	*ATG9, CCZ1, PEX12, PEX14, PEX19, PEX5, SLG1, SNF4, SNX4, TLG2, VPS1, VPS51*
Chromosome segregation	*CIK1, CTF18, CTF4, CTF8, DCC1, KAR3, MMS22, MRC1, RMI1, RTS1, SOH1*
Mitochondrion organization	*ATP11, COX12, COX23, FMC1, GEM1, GRS1, MMM1, MRPL36, NUM1, RIM1, TIM18*
Translation	*EAP1, GRS1, MRPL36, RPL21B, RPL35B, RPS11B, RPS16A, RPS18B, RPS4A, TEF4, TMA19*
Vesicle organization	*GOS1, SEC28, SSO2, TLG2, VAM3, VAM6, VAM7, VPS4, VPS41, VPS51*
Vacuole organization	*ATG9, CLA4, STE20, VAM10, VAM3, VAM6, VAM7, VPS41, YPT7*
Cellular carbohydrate metabolic process	*ELM1, FKS1, MAL12, OST4, PDC5, PFK2, SNF4, SOL4, TPS2*
Signaling	*CLA4, FAR1, MRC1, OPI1, PHO80, SLG1, STE20, TAX4*
Sporulation resulting in formation of a cellular spore	*MRE11, RIM21, RIM9, SSO2, UBI4, XRS2, YPL205C*
Cytoskeleton organization	*CIK1, GCS1, NUM1, SHE1, SLG1, VPS1*
Ribosome biogenesis	*DRS2, NOP12, RPL35B, RPS11B, RPS16A, RPS18B*
Cytokinesis	*CLA4, CTS1, CYK3, ELM1, STE20*
Generation of precursor metabolites and energy	*CYT1, ETR1, OAR1, PDC5, PFK2*
Cofactor metabolic process	*CAT5, SOL4, SPE1, SPE2, SPE3*
Cell budding	*CLA4, ELM1, MUB1, URM1, VPS51*
Cellular homeostasis	*MEH1, PHO80, SKY1, SPF1, ZRC1*
Conjugation	*CIK1, FAR1, KAR3, STE20*
Pseudohyphal growth	*DFG16, ELM1, SOK2, STE20*
Fungal-type cell wall organization	*ECM8, KRE1, SLG1, TAX4*
Heterocycle metabolic process	*AAH1, IMD3, PDC5, SOL4*
Cellular aromatic compound metabolic process	*AAH1, ARO1, ARO2, PDC5*
Cellular protein catabolic process	*DIA2, NPL4, UBX2, VMS1*
Cellular respiration	*CYT1, ETR1, OAR1*
Nucleus organization	*APQ12, CIK1, KAR3*
Vitamin metabolic process	*SPE1, SPE2, SPE3*
Cellular component morphogenesis	*ELM1, SSO2*
Transposition	*MMS1, RTT109*
Protein folding	*EGD2*

Table legend: *RAD52*-like genes in this study were analysed using SGD gene ontology slim mapper (http://www.yeastgenome.org/cgi-bin/GO/goSlimMapper.pl). “Yeast GO-Slim: Process” GO set was used to determine the list of genes that involved in different biological processes.
